# Growing gender disparity in HIV infection in Africa: sources and policy implications

**DOI:** 10.1101/2023.03.16.23287351

**Published:** 2023-03-20

**Authors:** Mélodie Monod, Andrea Brizzi, Ronald M Galiwango, Robert Ssekubugu, Yu Chen, Xiaoyue Xi, Edward Nelson Kankaka, Victor Ssempijja, Lucie Abeler Dörner, Adam Akullian, Alexandra Blenkinsop, David Bonsall, Larry W Chang, Shozen Dan, Christophe Fraser, Tanya Golubchik, Ronald H Gray, Matthew Hall, Jade C Jackson, Godfrey Kigozi, Oliver Laeyendecker, Lisa A. Mills, Thomas C. Quinn, Steven J. Reynolds, John Santelli, Nelson K. Sewankambo, Simon EF Spencer, Joseph Ssekasanvu, Laura Thomson, Maria J Wawer, David Serwadda, Peter Godfrey-Faussett, Joseph Kagaayi, M Kate Grabowski, Oliver Ratmann

**Affiliations:** 1 Department of Mathematics, Imperial College London, London, United Kingdom.; 2 Rakai Health Sciences Program, Kalisizo, Uganda.; 3 Division of Infectious diseases, Johns Hopkins School of Medicine, Baltimore, Maryland, United States.; 4 Research Department, Rakai Health Sciences Program, Rakai, Uganda.; 5 Clinical Monitoring Research Program Directorate, Frederick National Laboratory for Cancer Research, Frederick,Maryland, United States.; 6 Statistics Department, Rakai Health Sciences Program, Rakai, Uganda.; 7 Big Data Institute, University of Oxford, Oxford, United Kingdom.; 8 Bill and Melinda Gates Foundation, Seattle, Washington, United States.; 9 Division of Global HIV and TB, U. S. Centers for Disease Control and Prevention, Kampala, Uganda.; 10 Wellcome Centre for Human Genomics, Nuffield Department of Medicine, Univeristy of Oxford, Oxford, United Kingdom.; 11 Pandemic Sciences Institute, Univeristy of Oxford, Oxford, United Kingdom.; 12 Sydney Infectious Diseases Institute, School of Medical Sciences, Faculty of Medicine and Health, University of Sydney, Sydney, Australia.; 13 Big Data Institute, Nuffield Department of Medicine, University of Oxford, Oxford, United Kingdom.; 14 Professor Emeritus, Department of Epidemiology, Johns Hopkins University, Bloomberg School of Public Health, Baltimore, Maryland, United States.; 15 Department of Pathology, Johns Hopkins School of Medicine, Baltimore, Maryland, United States.; 16 Division of Intramural Research, National Institute of Allergy and Infectious Diseases, National Institutes of Health, Bethesda, Maryland, United States.; 17 Department of Medicine, Johns Hopkins School of Medicine, Baltimore, Maryland, United States.; 18 Population and Family Health and Pediatrics, Columbia Mailman School of Public Health, New York, United States.; 19 College of Health Sciences, School of Medicine, Makerere University, Kampala, Uganda.; 20 Department of Statistics, University of Warwick, Coventry, United Kingdom.; 21 Department of Epidemiology, Johns Hopkins Bloomberg School of Public Health, Baltimore, Maryland, United States.; 22 Department of Infectious and Tropical Diseases, London School of Hygiene & Tropical Medicine, London, United Kingdom.

## Abstract

HIV incidence in eastern and southern Africa has historically been concentrated among girls and women aged 15-24 years, but as new cases decline with HIV interventions, population-level infection dynamics may shift by age and gender. Here, we integrated population-based surveillance and longitudinal deep-sequence viral phylogenetics to assess how HIV incidence and the population groups driving transmission have evolved over a 15 year period from 2003 to 2018 in Uganda. HIV viral suppression increased more rapidly in women than men, resulting in 1.5-2 fold higher suppression rates in women with HIV by 2018 across age groups. Incidence declined more slowly in women than men, increasing pre-existing gender imbalance in HIV burden. Age-specific transmission flows shifted; the share of transmission to girls and women aged 15-24 years from older men declined by approximately one third, whereas the contribution of transmission to women aged 25-34 years from men aged 0-6 years older doubled from 2003 to 2018. We estimated closing the gender gap in viral suppression could have reduced HIV incidence in women by half in 2018 and ended gender disparities in incidence. This study suggests that male-targeted HIV programs to increase HIV suppression are critical to reduce incidence in women, close gender gaps in infection burden and improve men’s health in Africa.

## Introduction

Despite the widespread availability of HIV prevention and treatment interventions, there were 1.5 million new HIV infections and 680,000 HIV-associated deaths in 2020^1^. More than half of these new cases and deaths were concentrated in the eastern and southern regions of the African continent, where incidence rates have historically been highest in adolescent girls and young women, aged 15-24 years^2,3,4,5^. While HIV incidence has declined by 43% in eastern and southern Africa since 2010, current HIV service programs are failing to reduce new cases rapidly enough to meet United Nations health targets for HIV epidemic control and to keep the costs of life-long medical treatment manageable in the absence of a cure for HIV^1,6^. More broadly, uncontrolled HIV spread has far-ranging public health consequences beyond HIV-associated morbidity and mortality as shown by emerging data on extensive evolution of severe acute respiratory syndrome coronavirus 2 (SARS-CoV-2) among persons with HIV antiretroviral treatment failure^7,8^. With rising levels of HIV drug resistance^9,10^ and flatlined global investment in HIV control^11^, the African HIV epidemic has reached a critical inflection point^12,13,14,15^.

**Figure 1:**
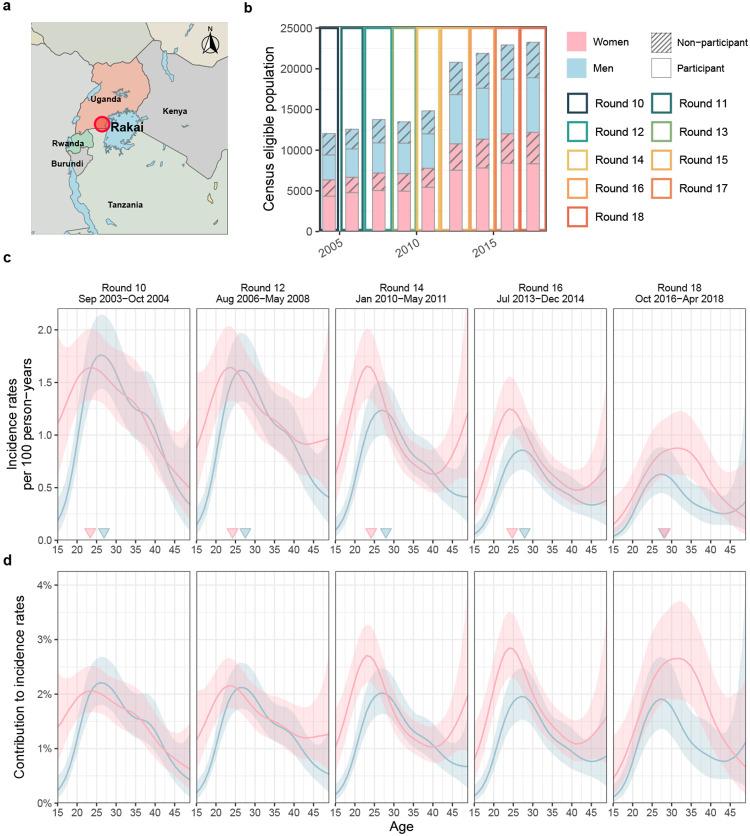
Time trends in age-specific HIV incidence rates for men and women in Rakai, Uganda. (**a**) Location of the Rakai Community Cohort Study (RCCS) in south-central Uganda. Study outcomes are reported for all RCCS communities located inland to Lake Victoria across nine survey rounds. (**b**) Number of RCCS participants in the census-eligible population of age 15 to 49 by survey round. (**c**) Estimated mean HIV incidence rates per 100 person-years of exposure in uninfected individuals (line) by 1-year age band, gender and survey round, along with 95% confidence intervals (ribbon), and median age of incident cases (cross). (**d**) Estimated contribution to incidence rates (line) by 1-year age band, gender and survey round, along with 95% confidence intervals (ribbon).

Efficient delivery of HIV interventions is arguably more important now than ever^5^. Over the last decade, African HIV control programs, including the United States President’s Emergency Plan for AIDS Relief (PEPFAR), have focused on expanding treatment coverage in people with HIV and reducing HIV infections among adolescent girls and young women^16,17^. However, recent data from Africa indicate that the mean age of infection is shifting^18,19^ and incidence rates are declining faster in men than in women^20,21^, suggesting that the age and gender structure of the African HIV epidemic is evolving. Here, we integrate 15 years of data on HIV incidence and onward transmission to show how the drivers of the African HIV epidemic are changing and how HIV services can respond to deliver sustained, fast, and universal reductions. We assessed how HIV transmission dynamics have evolved by gender and age from 2003 to 2018, and evaluated policy implications in counterfactual modelling scenarios, using multi-system data from the population-based Rakai Community Cohort Study (RCCS) in south-central Uganda^22^. The study population comprised men and women aged 15 to 49 years in 36 RCCS semi-urban and rural agrarian communities with an HIV risk profile typical across eastern and southern Africa^23,24^ (Figure 1a). We followed individuals in the RCCS who were HIV seronegative and documented new infection events. We also deep-sequenced HIV virus longitudinally from almost all persons with HIV and sufficient viral load for sequencing. This enabled us to measure directed transmission networks across age and gender as in other locations contributing to the PANGEA-HIV network^25,26,27,28,29^, but with a primary focus on the evolution of infection dynamics and transmission networks over time and during mass scale-up of HIV services in Africa^1^. These data provide detailed insights into the changing drivers of HIV transmission, which we interpret in the context of population-level HIV viral load, treatment uptake, and sexual behaviour surveys conducted in the same population. Table 1 summarizes our findings and policy implications.

## Results

### HIV incidence is declining faster in men than women

**Extended Data Fig. 1:**
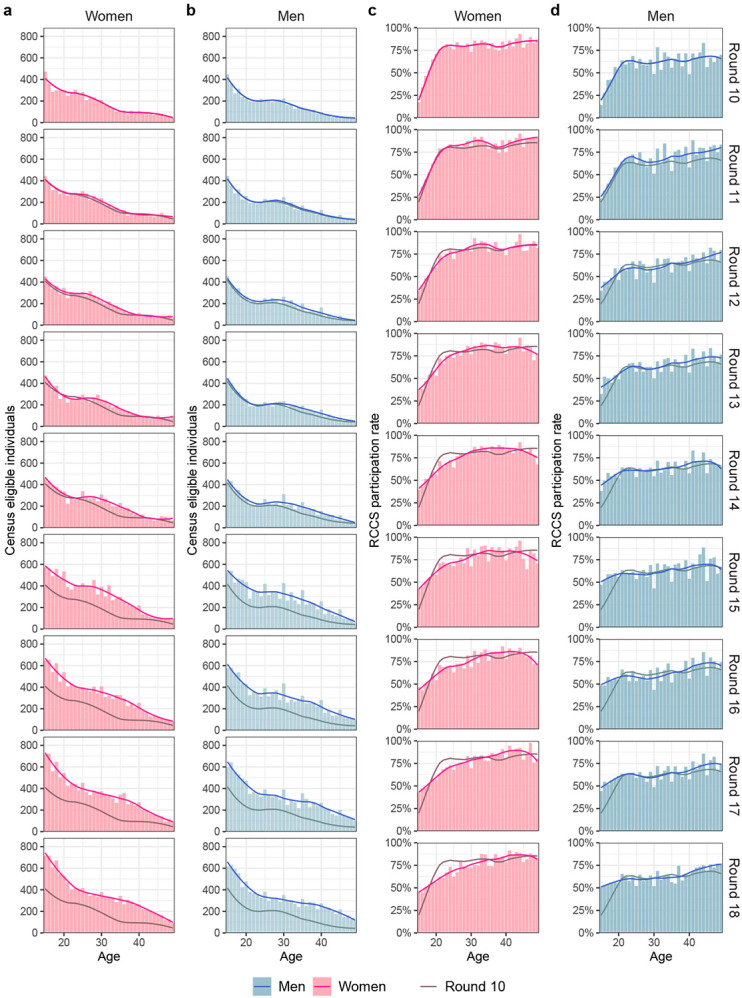
Characteristics of the RCCS study population by age, gender, and time. (**a-b**) Population size. Counts of the aggregated individual-level census data by 1-year age group (bars) are shown along LOESS smoothed population size estimates (line) for men and women (see text). (**c-d**) RCCS participation rates. Rates relative to the aggregated census data by 1-year age band (bars) are shown along LOESS smoothed participation rates (line) for both men and women. For reference, round 10 values are indicated in each subsequent plot in darker colours. The timeline of the survey rounds is shown in Figure 1b.

From September 23, 2003 to May 22, 2018, 36,988 participants were enrolled in the Rakai Community Cohort Study^20,30,31^. Of these participants, 22,729 tested HIV seronegative at first survey, and contributed an estimated 127,217 person-years of follow-up (Fig. 1b, Supplementary Table S1- S2). Study participants were enrolled following population census, household enumeration, and informed consent in 9 survey rounds of approximately 18 months duration, herein denoted as survey rounds 10-18 (see Methods). Between 67.8% and 72.6% of individuals who were present at time of survey participated. Most non-participants were absent for school or work outside their home communities, resulting in age- and gender-specific participation rates averaging 69% (Extended Data Fig. 1).

**Extended Data Fig. 2:**
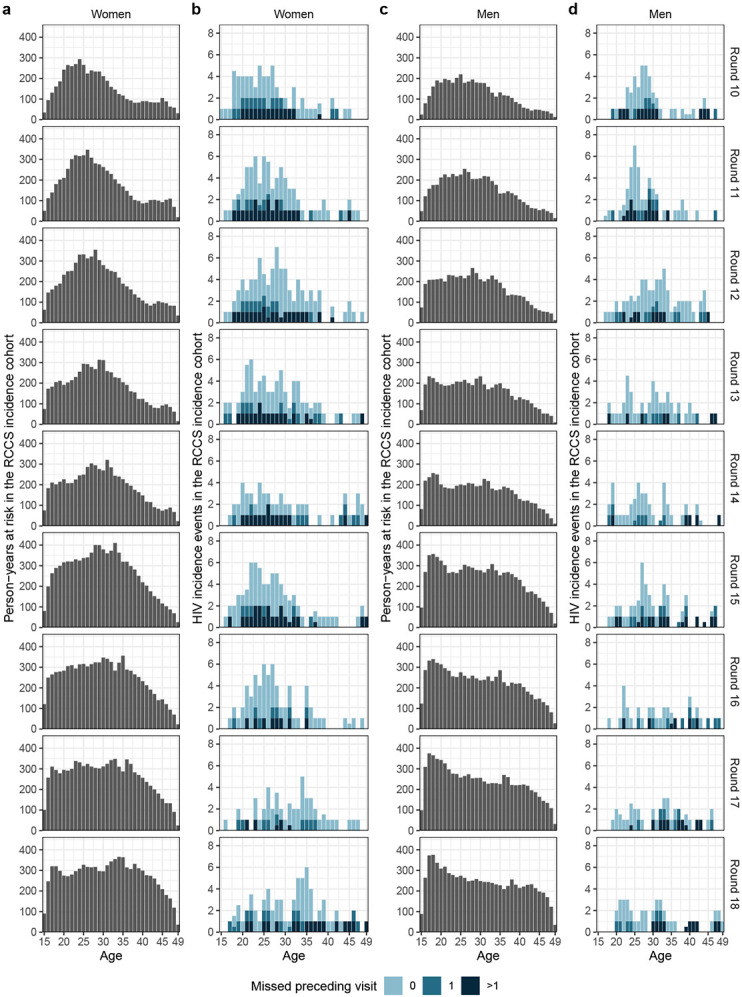
Age- and gender-specific person-years at risk and HIV incidence events in the RCCS incidence cohort. Person-years at risk in the RCCS incidence cohort among (**a**) women and (**c**) men. HIV incidence events in the RCCS incidence cohort among (**b**) women and (**d**) men.

**Extended Data Fig. 3:**
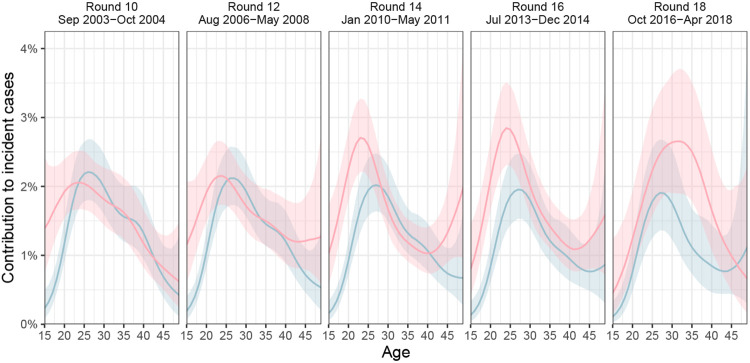
Age- and gender-specific contributions to HIV incident cases by round. Estimated contribution to incident cases in the study population (line) by 1-year age band, gender and survey round, along with 95% confidence intervals (ribbon).

In total, we observed 1,100 incident HIV infections (Supplementary Tables S3-S4 and Extended Data Fig. 2). Fig. 1c shows that incidence rates among men in inland communities fell rapidly from 1.05 [1.03-1.08] per 100 person-years (PY) in 2003 (survey round 10) by 67.8% [66.2-69.2] to 0.34 [0.33-0.35] per 100 PY in 2018 (survey round 18), with no substantial shift in the median age of male incident infection (blue triangles in Fig. 1c). In young women aged 15 to 24 years, incidence rates fell similarly rapidly from 1.42 [1.35-1.5] per 100PY in 2003 by 74.5% [71.6-77.1] to 0.36 [0.33-0.4] per 100PY in 2018. However, among women aged 25-34, declines in HIV incidence were substantially slower (from 1.51 [1.45-1.57] per 100PY in 2003 by 43.9% [40.5-47.4] to 0.84 [0.8-0.89] per 100PY in 2018), and similarly in women aged 35-49 (from 0.9 [0.85-0.94] per 100PY in 2003 by 37.4% [31.9-42.6] to 0.56 [0.52-0.6] per 100PY in 2018), resulting in a progressive, substantial shift in the median age of infection in women from 23.4 [22.6-24.1] in 2003 to 28.2 [27.1-29.2] in 2018 (Fig. 1c-d and Extended Data Fig. 3). Progress in reducing HIV incidence thus continues to be substantially slower in women^20,32,33^, especially among those aged 25 years and above.

### The proportion of transmission from men is increasing

**Extended Data Fig. 4:**
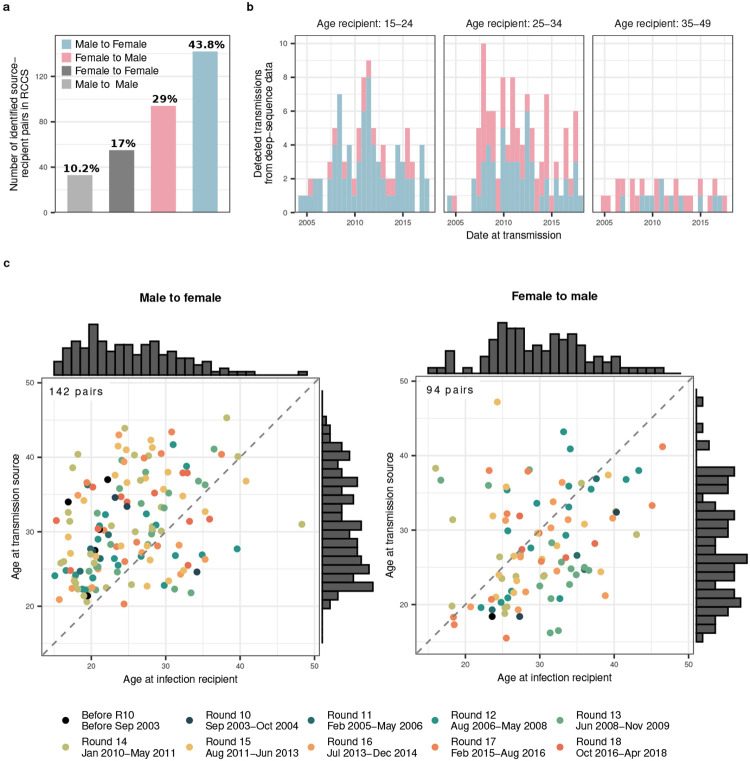
Phylogenetically reconstructed source-recipient pairs. (**a**) Number of source-recipient pairs by gender (colour). (**b**) Number of heterosexual source-recipient pairs by the date of infection of the recipient (x-axis), the age of the recipient at infection (panel), and transmission direction. (**c**) Heterosexual source-recipient pairs by the age of the source (x-axis) and the age of the recipient (y-axis) at the median infection time estimate by the round (colour) in which transmission was estimated to have occurred. The number of phylogenetically reconstructed source-recipient pairs is indicated in the top-left corner.

**Extended Data Fig. 5:**
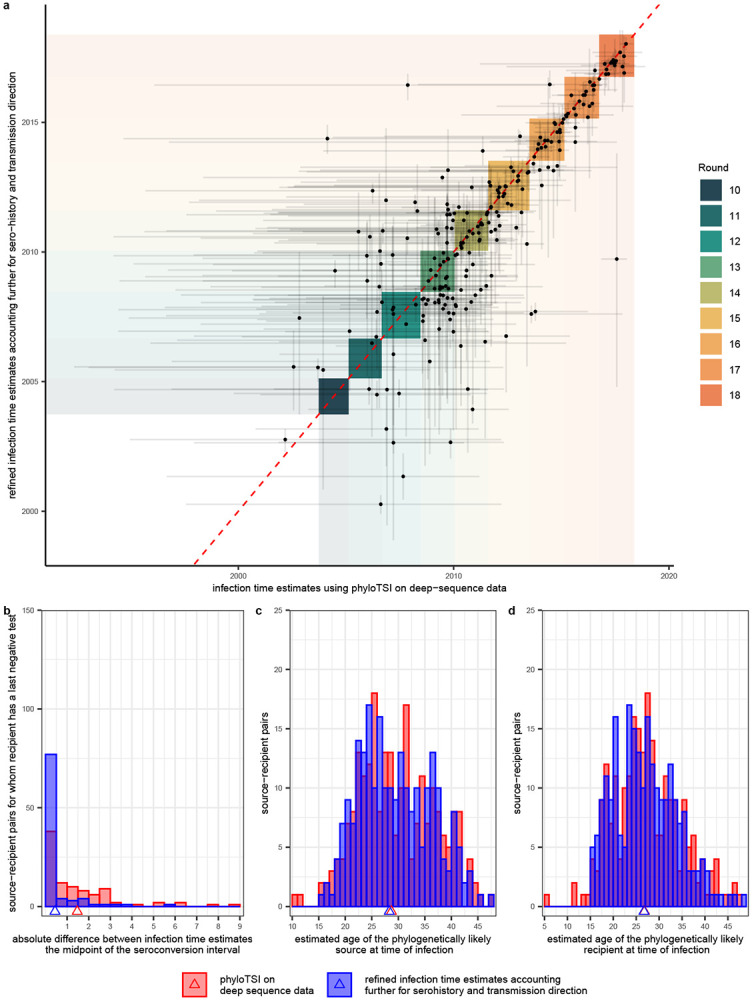
Comparison of estimated infection dates in phylogenetically reconstructed source-recipient pairs. (**a**) Estimated infection times of the recipient in the phylogenetically reconstructed source-recipient pairs from phyloTSI based on deep-sequence data alone (x-axis) against refined estimates accounting for serohistory and inferred direction of transmission (y-axis). Median estimates (dots) are shown along 95% uncertainty ranges (lines). (**b**) Histogram of absolute difference (bars) and mean absolute differences (triangle) between infection time estimates and the midpoint of seroconversion intervals in 98 source-recipient pairs in which the recipient had a last negative test, across the two methods (colour). (**c**) Histogram (bars) and median (triangle) age of the phylogenetically likely recipient, across the two methods (colors). (**d**) Histogram (bars) and median (triangle) age of the phylogenetically likely source, across the two methods (colors).

To characterize the population transmission flows by age and gender that underly observed shifts in incidence, we deep-sequenced virus from 2174 participants with HIV and sufficient viral load for sequencing (Supplementary Table S5^25^). By embedding genomic surveillance into a population-based cohort study, deep-sequence sampling coverage was high relative to typical pathogen sequencing studies, which is essential for reconstructing transmission events (Supplementary Table S6^29,34,35,36,37,38^). Deep-sequencing was performed from 2010 (survey round 14) onwards, but because sequences provide information on past and present transmission events, we also obtained information on transmission in earlier rounds and calculated sequence coverage in participants that were ever deep-sequenced at minimum quality criteria suitable for phylogenetic analysis (Supplementary Table S6). Overall, sequence sampling coverage of participants with HIV ranged between 46% and 56% from round 14 onwards. We next identified the phylogenetic ordering between multiple viral variants from individuals and estimate the direction of transmission with *phyloscanner* (Methods)^34,39^. We identified 236 heterosexual source-recipient pairs that were phylogenetically close and exhibited, in combination with data on last negative and first positive tests, strongly consistent evidence of the direction of transmission (Methods and Extended Data Fig. 4). We did not analyse further 55 female-female source-recipient pairs between whom transmission is biologically extremely unlikely^40^ and who likely represent pairs with an unobserved male transmitting partner, as well as 33 male-male source-recipient pairs (Extended Data Fig. 4). We further estimated the likely infection date from deep-sequence data (Methods^41^), which enabled us to place the source-recipient pairs in calendar time (Extended Data Fig. 5). Of the 236 heterosexual source-recipient pairs, we retained in total 227 pairs in whom transmission was estimated to have occurred during the study period.

**Extended Data Fig. 6:**
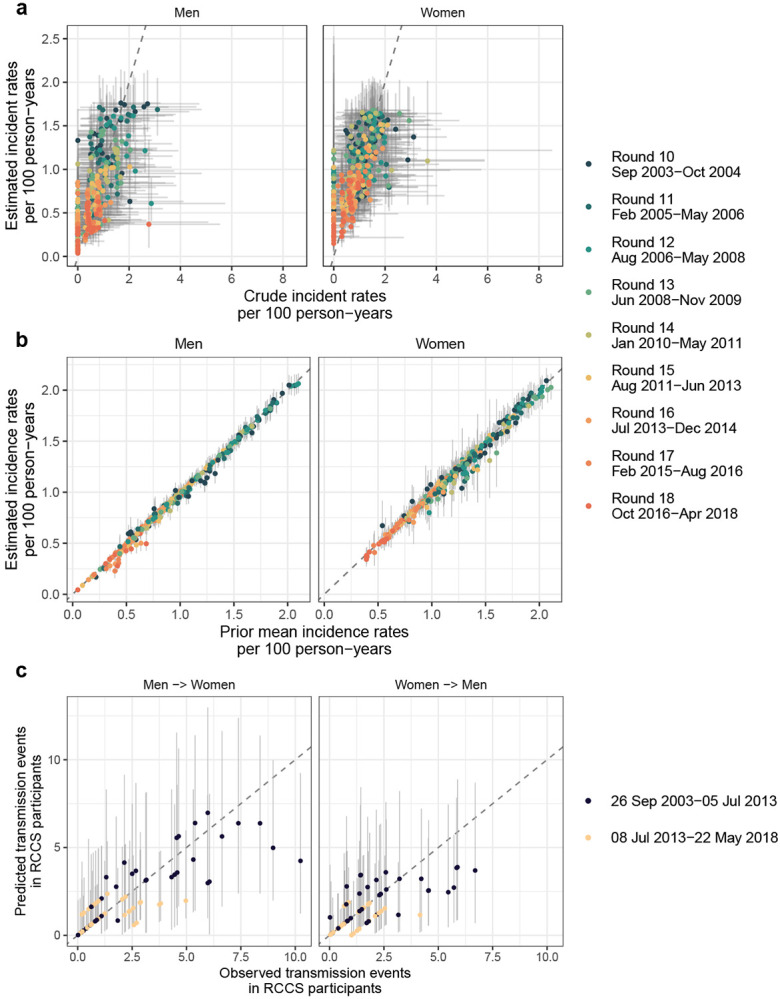
Validation of the incidence rate and transmission flow models. (**a**) Validation of the incidence rate model. To investigate model fit, empirical HIV incidence rates were obtained for each of the 50 data sets with imputed exposure times and compared to the estimated HIV incidence rates under the Poisson model. The median (point) and 95% range (horizontal error bars) of the crude HIV incidence rates are plotted against the posterior median (point) and 95% range (vertical error bars) of estimated HIV incidence rates for each gender (columns) and survey round (colour). (**b**) Validation of the transmission flow model. Median (point) and 95% credible interval (error bars) of the estimated incidence rates from the phylo-SIR model against the corresponding, informative prior means for each gender (columns) and survey round (colour) that were specified according to the outputs of the incidence rate model. (**c**) Posterior predictive check on the observed transmission flow counts by age of the phylogenetically likely source. Median (point) and 95% credible interval (error bars) of the predicted flow counts against the data for each gender-specific direction (columns) and time period (colour).

**Figure 2:**
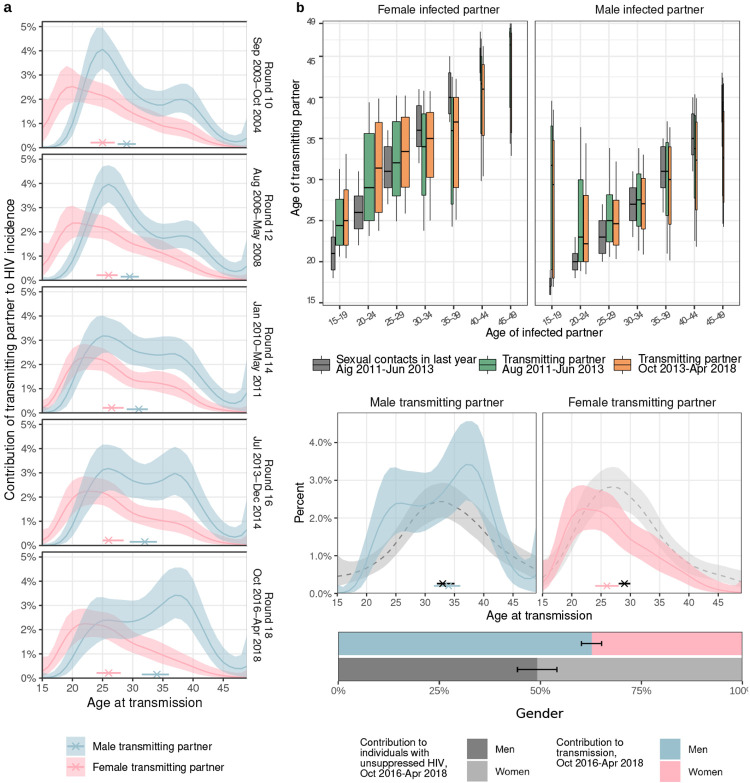
Time trends in age-specific sources of HIV infections in women and men. (**a**) Estimated age distributions of transmitting partners (posterior median: line, 95% credible interval: ribbon), along with the median age at transmission (posterior median: cross, 95% credible interval: linebar). Age contributions sum to 100% for each round, suimning over men and women. (**b**) Estimated age distributions of transmitting partners by 5-year age bracket of infected partners. Boxplots show posterior median estimates, the posterior 50% interquartile range, and 80% credible intervals. The width of the boxes is proportional to the total infections in each recipient group. For reference, posterior estimates of the age distributions of sexual contact partners of men and women by 5-year age bands in the past 12 months in the same communities are shown in dark grey. (**c**) Comparison of the age contributions to transmitting partners (colour) to the age contributions to men and women with unsuppressed HIV (posterior median: dashed black line, 95% credible interval: ribbon), along with median age (posterior median: cross, 95% credible interval: linebar). Age contributions sum to 100% for men and women combined.

Deep-sequence phylogenetics cannot prove direction of transmission between two persons^34,42,43^, but in aggregate these data are able to capture HIV transmission flows by age and gender over time at a population level^29,44^. To interpret deep-sequence data, we modelled the phylogenetic sampling frame by quantifying the detection probability of incident HIV infections by age and gender. We then estimated population-level transmission flows adjusting for detection probabilities with semi-parametric Poisson flow regression models^45^, and under the constraint that the transmission flows needed to closely match the age- and gender-specific incidence dynamics shown in Fig. 1 (Methods^29^). The fitted model was consistent with all the available data (Extended Data Fig. 6). Fig. 2a shows the age profile of the estimated male and female sources of infection, such that the male plus the female sources sum to 100% for each survey round. Overall, we found that the contribution of men to onward transmission increased progressively from 57.9% [56.1-59.6] in 2003 to 62.8% [60.2-65.2] in 2018, indicating that HIV transmission is now more disproportionely driven by men than has been the case previously.

### Transmissions from men are shifting to older ages

The age profile of the population-level sources of infection characterizes the major age groups that sustain transmission^46^. We find that the age of transmitting male partners progressively increased from a median age of 28.7 [27.1-30.1] years in 2003 to 33.3 [31.0-35.7] years in 2018 (Table 2 and Fig. 2a), and this increase in the age of transmitting male partners was largest in transmissions to women aged 20-24 (Fig. 2b and Supplementary Table S9). In contrast, the median age of female transmitting partners remained similar (from 25.0 [23.0-27.0] years in 2003 to 26.0 [24.0-28.0] years in 2018), corresponding to our earlier observations that the age of male incident infections also remained similar during the observation period (triangles in Fig. 1a).

Over time, substantially fewer infections occurred in adolescent girls and young women aged 15-24 years. In 2003 the largest transmission flows were to women aged 15-24 years from male partners 0-6 years older (16.0% [12.8-19.3]) and from male partners 6+ years older (15.5% [12.2-18.8]), with a similar share of transmission flows in 2012. By 2018, these transmission flows declined by approximately one third, with 8.7% [6.2-11.7], to women aged 15-24 years from male partners aged 0-6 years older, and 11.5% [8.6-14.7] to women aged 15-24 years from male partners aged 6+ years older. In those infections in adolescent girls and young women that occurred in 2018, the median age difference between incident infections in adolescent girls and young women and their transmitting male partners were 9.0 [6.0-11.0] years (Fig. 2b and Supplementary Table S9). These age discrepancies in transmitting male partners and adolescent girls and young women are similarly large as in a phylogenetic study from KwaZulu-Natal in South Africa^47^. This prompted us to estimate for comparison age-specific sexual contact patterns within RCCS communities from data on the number and age of sexual partners of study participants with and without HIV (Methods). In 2018, the median age difference between adolescent girls and young women and their male sexual partners was 3.6 [3.5-3.9] years. Our data thus indicate that the main transmission flow into adolescent girls and young women is through contacts with considerably older men as compared to their typical sexual contacts^47,48^, and that while this transmission flow has weakened overall, it remains the predominant mode of infection in adolescent girls and young women.

By 2018, the largest share of transmission flows shifted to women aged 25-34 years, from male partners 0-6 years older. In 2003, transmissions to women 25-34 years from these transmitting partners accounted for 8.5% [7.0-10.2] of all transmissions, and by 2018 the share of these flows doubled to 13.2% [10.5-16.0] (Supplementary Table S9). We also find that the transmission flows to women aged 35 years and above increased (Supplementary Table S9, also indicated by wider boxplots in Fig. 2b).

**Extended Data Fig. 7:**
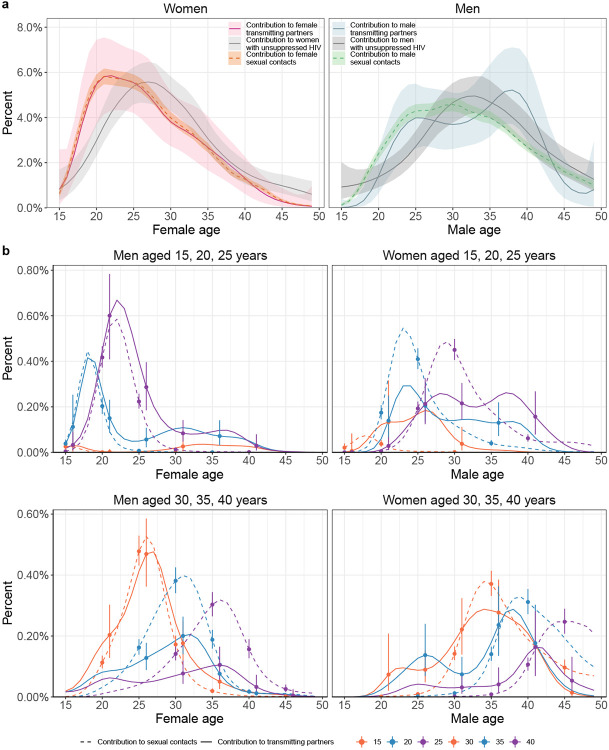
Age contributions to sexual contacts, viral suppression and transmission. (**a**) Estimated age contributions from women to men of all ages (left) and from men to women of all ages (right) to sexual contacts in round 15, viral suppression in round 18, and transmission in round 18 (posterior median: line, 95% credible interval: ribbon). Age contributions sum to 100% separately for women and men. (**b**) Estimated age contributions from women to men of specific ages (left) and from men to women of specific ages (right) to sexual contacts in round 15, and transmission in round 18 (posterior median: line, 95% credible interval: errorbars). Age contributions sum to 100% for women and men combined.

**Figure 3:**
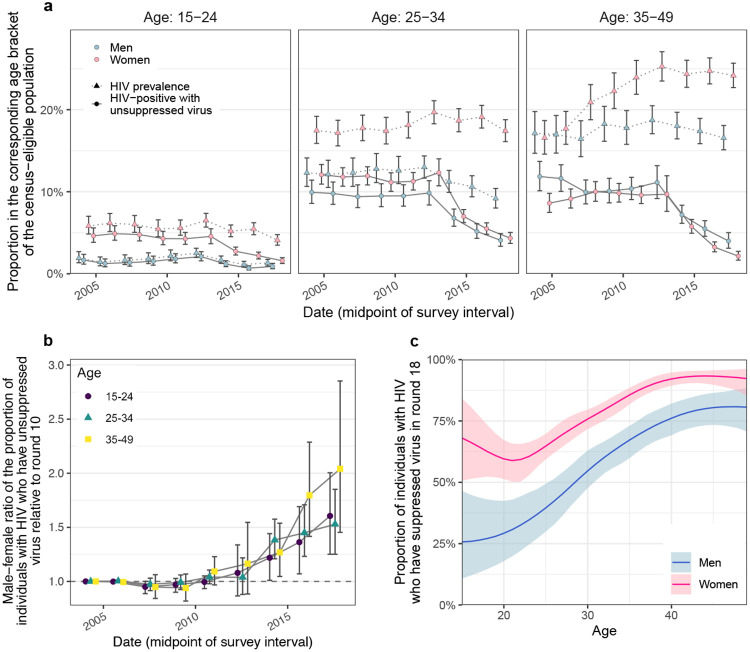
Changes in population-level suppression of HIV viral load. (**a**) Estimated trends in HIV prevalence and the proportion of census-eligible individuals in three age brackets that remain virally unsuppressed, defined as viral load above 1,000 copies/mL blood (posterior median: dots, 95% credible interval: errorbars), combining data from participants and from first-time participants as proxy of non-participants. (**b**) Male-to-female ratio in changes in population-level viral load suppression relative to round 10 (posterior median: dots, 95% credible interval: errorbars). (**c**) Estimated viral suppression rates by 1-year age band (x-axis) and gender (colour) for survey round 18. Throughout, estimates are based on data from participants and data from first-time participants as proxies of individuals who did not participate in the survey.

Our data suggest further deviations in age-specific transmission flows from the typical sexual contact patterns within study communities. For all women aged 30 years and older, we estimate their male transmitting partners were of similar age with a posterior interquartile age range of 32.1-38.2 years in 2018, whereas for comparison the typical sexual contact partners of these women were older with a posterior interquartile age range of 40.0-42.7. These findings explain the unexpected age profile of male transmitting partners (Fig. 3c) that concentrates in men aged 25 to 40 instead of extending to progressively older men (Extended Data Fig. 7b). These observations are in line with recent studies from Zambia^29^ and South Africa^49^ that show having a male partner aged 25-40 years rather than the age gap between partners is associated with increased transmission risk.

The transmission flows into men remained similar over time (Fig. 2b). In 2018, the largest transmission flow was to men aged 25-34 years from transmitting female partners of similar age that were 0-6 years older and accounted for one third of all transmission into men (11.0% [9.3-12.6]). Men in their twenties tended to acquire HIV from women with unusually small age difference as compared to the typical age differences with their sexual contact partners (Fig. 2b). For example, the median age difference between incident infections in men aged 20-24 years and their female transmitting partners was 0.0 [(−3.0)-2.0] years in 2018 while the median age difference between all men aged 20-24 years and their female sexual partners was 2.4 [2.2-2.4] years in 2018.

### Gender gaps in population-level viral suppression are increasing

**Extended Data Fig. 8:**
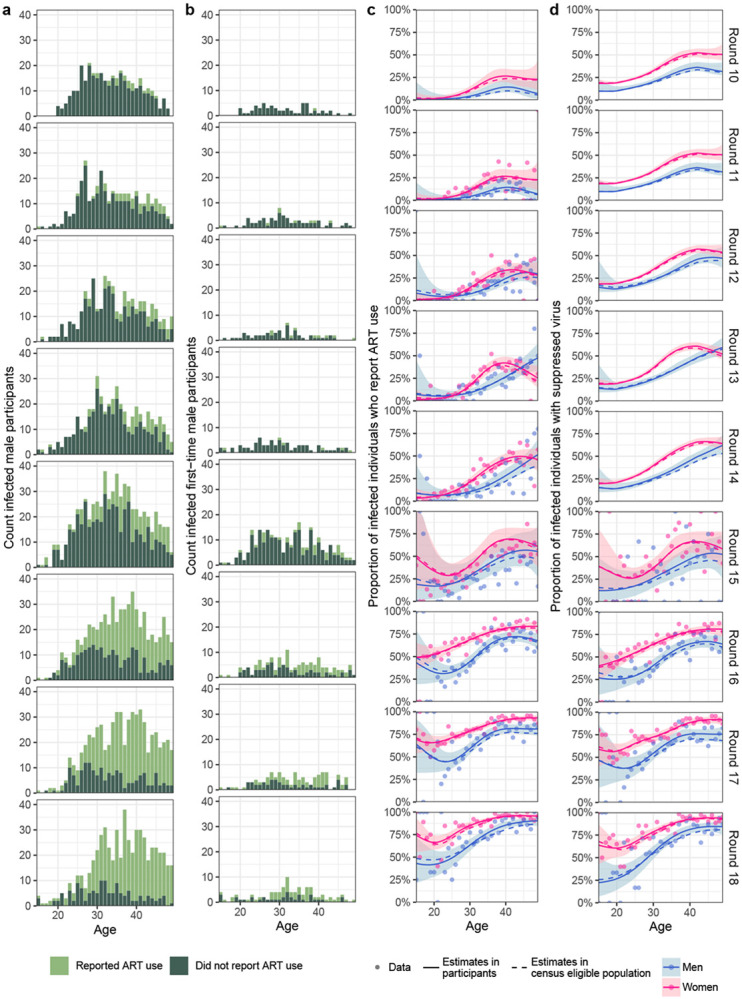
ART use and virus suppression in the RCCS study population by age, gender, and time. (**a**) HIV-positive male participants reporting ART use, by 1-year age band (x-axis) and survey round (rows). (**b**) HIV-positive male first-time participants reporting ART use, by 1-year age band (x-axis) and survey round (panel). (**c**) Estimates of ART use in men (blue) and women (pink) in the study population by 1-year of age. Data from participants (dots) are shown along smoothed posterior median estimates (solid line) and 95% credible intervals (ribbon) in participants, and along posterior median estimates in the census-eligible population (dashed line), using data from first-time participants as proxy of ART use in non-participants (see text). (**d**) Estimates of virus suppression, defined as a viral load measurement below 1,000 copies of HIV per milliliter plasma blood, in men (blue) and women (pink) in the study population by 1-year of age. Data and estimates shown as for ART use.

**Extended Data Fig. 9:**
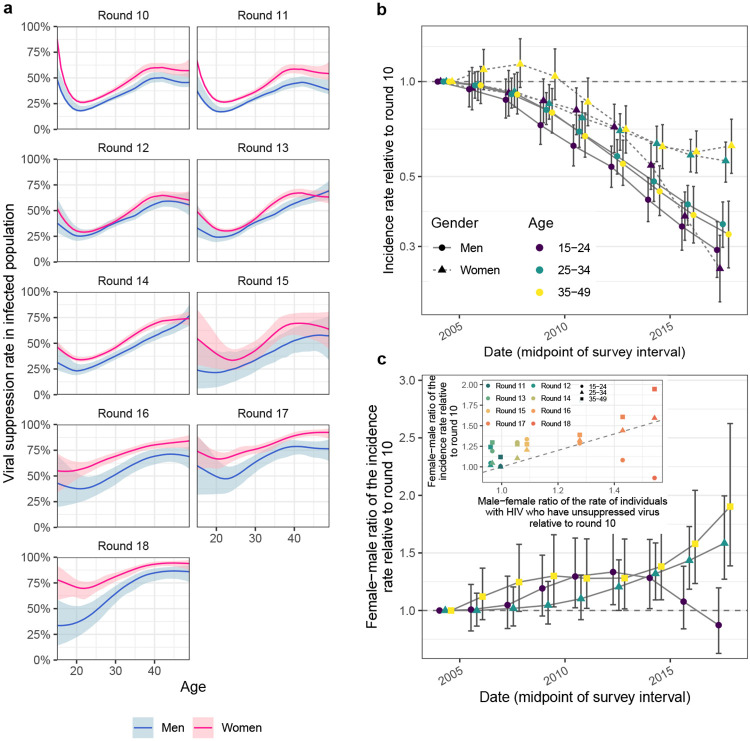
Comparison of age-specific viral suppression rates in census eligible men and women, 2003 to 2018. (**a**) Smoothed estimates of viral suppression rates defined as viral load below 1,000 copies/mL blood are shown by 1-year age band (x-axis), for each survey round (panel) and gender (colour). Estimates until round 15 are in part based on self-reported ART use data, and using data from first-time participants as proxies of individuals who did not participate in the survey. (**b**) Changes in incidence rates relative to round 10 (posterior median: dots, 95% credible interval: errorbars). (**c**) Female-to-male ratio in changes in incidence rates relative to round 10 (posterior median: dots, 95% credible interval: errorbars). The inset shows the correlation between faster declines in incidence in men and faster declines in population viral load in women.

We next placed the reconstructed shifts in transmission dynamics into the wider context of rapidly expanding HIV treatment during the observation period^20^. We measured viral load from 2011 (survey round 15) among almost all participants with HIV (Supplementary Table S1 and Extended Data Fig. 8)^50^. Following WHO criteria^51^, individuals with viral load measurements below 1,000 copies/millilitre (mL) plasma were considered virally suppressed (Methods). On average, 93% of individuals reporting ART use also had suppressed virus (Supplementary Table S7), leading us to estimate the number of individuals with suppressed virus before 2011 from corresponding ART use data (Methods). By 2018, we find that the proportion of men and women who have unsuppressed viral load was entirely decoupled from HIV prevalence in that while the proportion of women with HIV was substantially higher than in men, the proportion of women with unsuppressed viral load was similar or lower than in men (Fig. 3a). We quantified these trends with the male-to-female ratio of the proportion of virally unsuppressed individuals relative to 2003 levels, which has been progressively increasing in all age groups (Fig. 3b). This suggests^52^ that faster rises in female HIV suppression could explain in part the faster declines in male incidence rates as higher rates of ART uptake and virus suppression in women mean that male partners are less likely to become infected, whereas men’s higher rates of unsuppressed virus mean they are more likely to transmit to female partners than in the past (Extended Data Fig. 9). These trends have by 2018 accumulated to a substantial gap in suppression rates in men compared to women. At age 20, 59.5% [51.4-67.2] of women with HIV had suppressed virus compared to 29.2% [17.9-42.4] in men with HIV; while at age 30, this was 75.8% [71.7-79.7] in women with HIV versus 54.7% [47.6-63.0] in men with HIV; and at age 40 this was 92.1% [89.3-94.8] in women with HIV versus 76.1% [69.2-82.1] in men with HIV (Table 2 and Fig. 3c).

### Men contribute more to transmission than population viral load suggests

Combining phylogenetics with the virus suppression data also allowed us to compare transmission with population-level infectiousness as measured through individuals with unsuppressed virus (Table 2 and Fig. 2c). In 2018, the contribution of men to unsuppressed viral load was (49.2% [44.3-54.1]). For the same time period we found that the contribution of men to transmission was consistently higher (62.8% [60.2-65.2]), indicating that men contribute more to transmsission than population-viral load suggests. These findings are compatible with generally higher viral load in men than women^50,53^ that are expected to lead to higher transmission rates per sex act from men than women, heterogeneous contact patterns^54^, higher biological susceptibility of women to HIV infection^55,56^, but also lower susceptibility of men to HIV infection following voluntary medical male circumcision^31^.

### Closing the suppression gap in men could avert half of infections in women

**Figure 4:**
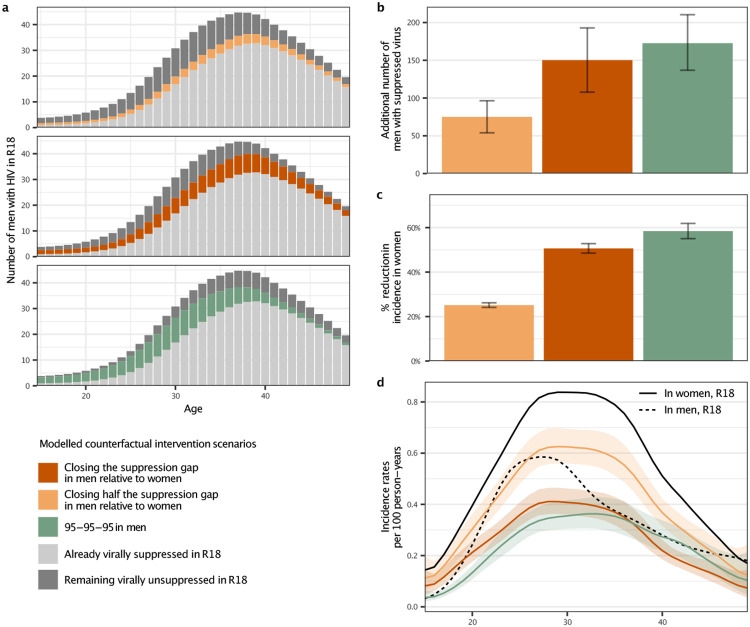
Counterfactual modelling scenarios predicting the impact of male-targeted interventions on incidence reductions in women. (**a-b**) Estimated additional number of men with HIV in the census-eligible population in round 18 that already had suppressed virus (light grey), are targeted in the counterfactual intervention scenarios (colour), and those who would have remained with unsuppressed virus in the counterfactuals (dark grey). (**c**) Percent reduction in incidence in women of the census-eligible population in round 18 under the counterfactual male-targeted scenarios. (**d**) Estimated incidence rates among women in the census-eligible population in round 18 (black solid line) and the counterfactual scenarios (colour), with incidence rates among men in round 18 shown as reference (black dashed line). Posterior median estimates are shown as lines, and 95% credible intervals as ribbons.

It is now well demonstrated that people with HIV who are on ART and maintain suppressed virus do not transmit HIV^57,58^. On this basis, we quantified the impact that closing the gap in male-female virus suppression rates could have had on the reconstructed, evolving HIV transmission flows. Specifically, we parameterised the semi-parametric Poisson transmission flow model in terms of HIV seronegative individuals who are susceptible to infection and individuals with unsuppressed HIV who remain infectious. Thus, we could use the fitted model to estimate the impact of fewer individuals with unsuppressed HIV on evolving HIV transmission in counterfactual, modelled intervention scenarios (see Methods). We considered the impact of three hypothetical scenarios: first, the impact of reducing by half the gap in the proportion of men with suppressed virus as compared to women (“closing half the suppression gap in men”) at the end of the observation period in 2018 (Fig. 3c); second, the impact of achieving the same virus suppression rates in men with HIV as in women in 2018 (“closing the suppression gap in men”); and third—for reference—achieving the UNAIDS 95-95-95 target that 86% of men (0.95 * 0.95 * 0.95) with HIV reach viral suppression in all age groups in 2018^59^. Table 2 and Fig. 4a describe the age-specific male counterfactual viral suppression targets of each scenario, and place these into the context of prevalence, suppression, and transmission. Overall, the closing the suppression gap scenarios targeted slightly older men compared to the age groups contributing to transmission from men, while the UNAIDS 95-95-95 scenario targeted slightly younger men . We predict that in the UNAIDS 95-95-95 scenario, an additional 172.6 [136.8-210.0] men with HIV would have reached viral suppression in 2018 (Fig. 4b) and this would have resulted in a 58.4% [55.1-61.6] additional reduction in HIV incidence in women in 2018 (Fig. 4c), which is in good agreement with the contribution of 95-95-95 interventions to projected incidence reductions for all of Eastern and Southern Africa under the mathematical models used to inform the global HIV prevention strategy^60^. In the scenario closing half the suppression gap in men, an additional 75.1 [53.9-96.0] men with HIV would have reached viral suppression in 2018 and resulted in a 25.2% [24.2-26.2] additional reduction in HIV incidence in women in 2018. In the scenario closing the entire suppression gap in men, an additional 150.2 [107.8-193.0] men with HIV would have reached viral suppression in 2018 and resulted in a 50.6% [48.7-52.7] additional reduction in HIV incidence in women in 2018 (Fig. 4b-c). Thus, all three male-targeted intervention scenarios involved reaching a small additional number of men compared to the thousands of women with higher risk of HIV acquisition in the same rural or semi-urban study areas^61^, while the scenarios aimed at closing the male-female suppression gap place greater emphasis on restoring gender equality in reaching and maintaining HIV suppression. Notably, given the disproportionally higher contribution of men to transmission relative to unsuppressed viremia (Table 2 and Fig. 3c), we predict that closing the suppression gap in men would have changed the female-to-male incidence rate ratio from 1.59 [1.38-1.82] to 0.77 [0.69-0.87] in 2018 (Fig. 4d), entirely closing the growing gender disparity in HIV incidence.

## Discussion

Effective HIV interventions and services are essential to bring most African countries on track to end AIDS as a public health threat by 2030 and accelerate progress towards the vision of the UNAIDS “three Zeros” target: zero new HIV infections, zero discrimination, and zero AIDS-related deaths^60,62^. Gender inequalities are among the main reasons why global targets on mass scale-up of HIV testing, biomedical interventions and on incidence reductions have not been achieved^63^. The new Global AIDS Strategy (2021-2026)^59^ is thus focused on maximising equitable and equal access to HIV services, and breaking down barriers to achieving viral suppression and preventing infection. However, there exists very little data on how the major disparities in HIV transmission in Africa have evolved over the past decade and what the policy implications might be. Here, we combined population-based incidence with deep-sequence viral phylogenetic surveillance data to characterize how HIV incidence and transmission sources have been evolving by age and gender in a typical rural to semi-urban African setting. We show that along with increasing availability of HIV services, there have been consistently faster increases in viral suppression in women than men. Viral suppression rates in women compared to men of the same age were 1.5 to 2 fold higher by 2018. In parallel, HIV incidence rates declined significantly faster among men than women. We demonstrate that an increasing majority of new infections are arising from men, thus widening the gender disparities in HIV burden that have existed in Africa for decades^4^. We also document substantial age shifts in HIV incidence and transmission sources, with the primary burden of incidence shifting to older women aged 25-34 years, the primary burden of transmission shifting to male partners aged 30-39 years, the relative contribution of transmission flows to adolescent girls and young women from older men reducing by one third, and the relative contribution of transmission flows to women aged 25-34 years from male partners 0-6 years older doubling by 2018. Modelling counterfactual improvements in HIV outcomes for men on the inferred transmission flows during the last survey round in 2016-2018, we find that closing the male gender gap in viral suppression rates could have reduced incident female infections by half in that time period and brought about gender equality in HIV infection burden. To reach the Three Zeros, it will be key for African HIV programs to address increasing gender disparity in viral suppression and transmission, disrupt evolving age-specific transmission flows, and prevent a growing proportion of new infections among older women aged 25-34 years from similarly aged men.

This study evaluated data from one longitudinal surveillance cohort in southern Uganda, but the increasing gender disparities and shifts in age-specific transmission are not unique. Empiric incidence data published over the last decade documents widespread declining incidence across the African continent^24^, and UNAIDS mathematic models from eastern and southern Africa have estimated a 43% reduction in incidence regionally since 2010^1^. Our findings are also compatible with gender-stratified HIV incidence data from prospective African population-level studies that indicate greater differences in rates of new infections between men and women in the same cohort over calendar time^24^, data from population surveillance studies and HIV treatment and prevention trials showing lower levels of viral suppression among men compared to women with HIV^64,65^, and phylogenetic studies from Botswana^28^ and Zambia^29^. Together, these observations suggest that the principal characteristics of the evolving HIV epidemic summarized in Table 1 likely apply more broadly in similar rural and semi-urban populations across Eastern and Southern Africa.

Given that the African HIV epidemic has historically been concentrated among adolescent girls and young women^4,5^, programs and policies rightfully have concentrated on reducing HIV risk in this demographic. But despite the substantially higher burden of HIV in women, we document here that most onward transmission is driven by men and that — as incidence is declining — the contribution of men to onward transmission is growing, likely due to lower levels of viral suppression in men and slower population-level declines in HIV viremia in men.

While there are emerging efforts to design male-centered HIV interventions^66,67,68^, African men continue to be overlooked in the design of programmatic services. For instance, the United States President’s Emergency Plan for AIDS Relief (PEFPAR) provides HIV prevention, care and treatment to most of the highest HIV burdened countries globally, including Uganda^69^. In 2022, PEFPAR released its strategic direction to end the AIDS epidemic by 2030, largely focusing on the prevention of HIV infections among adolescent girls and young women^17^. Many factors, including gender norms, mobility, and lack of targeted programming to men-contribute to lower uptake of HIV services by men^68^. Case finding of men with HIV might be difficult but could be strengthened by expanding access to HIV testing services most likely to reach them, such as through self-testing or assisted partner notification and other social network strategies^70,71,72^. Retention of men with HIV in treatment and care programs could be improved through male-centered differentiated service delivery. It is well-established that improving male engagement in HIV services leads to better health for men^73,74^. Critically, our data suggest further that had there been additional efforts to reach and maintain men in treatment services with HIV suppression at levels corresponding that of their female counterparts, half of new infections in adolescent girls and women could have been averted.

Our study has several strengths. Our findings are grounded in fifteen years of consecutive population-based epidemiologic and molecular surveillance in southern Uganda, enabling us to measure the changes in HIV incidence and transmission during a critical period of HIV service scale-up. Unlike other studies recently reporting on trends in HIV incidence in Africa^19,74^, we also collected HIV deep-sequence phylogenetic information to measure transmission networks with data on the direction of transmission, and directly relate shifts in HIV incidence to evolving transmission flows. Though it is typically assumed that age-specific patterns in onward HIV transmission correspond to those of viremia or follow typical sexual contact patterns, we find that this is not always the case. First, men contributed disproportionally more to onward transmission than to viraemia across all survey rounds during which viraemia were measured (Fig. 2c and Extended Data Fig. 7a). Second, older women contributed less to transmission than viraemia suggest, an observation that was consistent with attenuating sexual activity of women from age 25 onwards (Extended Data Fig. 7a). Third, young women and young men tended to be infected by transmitting partners who were substantially older than the typical sexual partners of the same population age group (Fig. 2b and Extended Data Fig. 7b). These findings illustrate the central utility of pathogen genomics to track and understand patterns of transmission, especially when interpreted in the context of population-based surveillance data, and when implemented at high enough sequence coverage to reconstruct directed transmission networks.

This study has important limitations. First, not all census-eligible individuals participated in the survey, primarily due to absence for work or school (Extended Data Fig. 1). We used data from first-time participants as proxies of non-participants, but we cannot rule out that non-participants include disproportionally larger populations of people living with HIV and/or with different risk profiles. Several sensitivity analyses (Supplementary Table S11) indicate that more men with unsuppressed HIV would have to be reached for HIV incidence reductions in women to do similar to the reductions in Figure 4. Second, we were unable to deep-sequence virus from individuals with suppressed HIV, and over time an increasing proportion of participants had already suppressed virus at the time of first survey visit (Extended Data Fig. 8). We do not know the impact of decreasing sequence coverage over time on our analyses. However^75^, we note that by integrating data from the prospective incidence cohort into our transmission flow analysis, we are able to adjust for sampling and censoring biases associated with retrospective reconstructions of transmission events, and to estimate population-level transmission flows that match longitudinal, age- and gender-specific incidence trends (Supplementary Table S9). Third, over time some communities were added and others left the Rakai Community Cohort Study (see Table S2). We repeated our analysis on the subset of 28 continuously surveyed communities, and found similar incidence and transmission dynamics with slightly faster declines in male new infections and larger gender disparities, although uncertainty ranges generally overlapped (Supplementary Table S11 and Supplementary Figure S3). Fourth, our findings on rural and semi-urban populations may not extend to populations with different demographics, risk profiles or healthcare access, and this includes populations in urban or metropolitan areas or key populations.

This study demonstrates shifting patterns in HIV incidence and in the drivers of HIV infection in East Africa, providing key data for evidence-informed policy making. We find incidence rates have dropped substantially in women aged 15-24 years from 2003 to 2018, and incidence rates now peak among women aged 25-35 years, consistent with cross-sectional national surveillance data from Uganda^6^. Long-term viral genomic surveillance shows that functionally, shifts in women’s incidence are the result of an increase in the age of transmitting male partners, and the primary contribution to HIV transmission lies now in men aged 30 and above. Gender disparity in HIV transmission is increasing, and the growing contribution of men to transmission is associated with substantially slower declines of unsuppressed viraemia in men than women, disproportional transmission risk per unsuppressed male partner, and unbroken transmission flows from unusually old or unusually young transmitting partners relative to typical sexual contact patterns. We predict successful interventions centred on men that bring suppression rates in men on par with those in women could reduce incidence in women by half and close the gender gap in new infections. These findings reinforce calls for HIV prevention programming and services to give greater priority to reach and retain in care men with HIV as this will improve male health, substantially reduce incidence in women, and close gender gaps in infection burden.

## 1 Online Methods

### Rakai Community Cohort Study

#### Longitudinal surveillance.

Between September 2003 and May 2018, nine consecutive survey rounds of the Rakai Community Cohort Study (RCCS) were conducted in 36 inland communities in south-central Uganda (Fig. 1 and Tables S1-S2). The results presented in this paper derive from data collected through these surveys, including the population census, the RCCS survey participants, the incidence cohort, and the phylogenetic transmission cohort.

RCCS survey methods have been reported previously^20,22^. In brief, for each survey round, the RCCS did a household census, and subsequently invited all individuals that were of age 15-49 years and residents for at least 1 month to participate in the open, longitudinal RCCS survey. Eligible individuals first attended group consent procedures, and individual consent was obtained privately by a trained RCCS interviewer. Following consent, participants reported on demographics, behavior, health, and health service use. All participants were offered free voluntary counseling and HIV testing as part of the survey. Rapid tests at the time of the survey and confirmatory enzyme immunoassays were performed to determine HIV status. All participants were provided with pre-test and post-test counseling, and referrals of individuals who were HIV-positive for ART. Additionally, all consenting participants, irrespective of HIV status, were offered a venous blood sample for storage/future testing, including viral phylogenetic studies. Table S1 summarises the characteristics of the RCCS participants and HIV-positive participants by age and gender. For the purpose of our analyses, we combined data from three pairs of geographically close areas in periurban settings into three communities, and 28 of 36 communities were continuously surveyed over all rounds (Table S2).

#### Population size estimates.

To characterise changes in population demography, individual-level data on the census-eligible individuals that were obtained during each census were aggregated by gender, 1-year age band (between 15 and 49 years) and survey round (Extended Data Fig. 1a-b, bars). The reported age in the census surveys tended to reflect grouping patterns towards multiples of 5, suggesting that individuals reported their age only approximately. For this reason, we smoothed population sizes across ages independently for every gender and survey round, using locally weighted running line smoother (LOESS) regression methods that fit multiple polynomial regressions in local neighborhoods as implemented in the R package stats version 3.6.2 with span argument set to 0.5 (Extended Data Fig. 1a-b, line).

#### Participation rates.

To characterise participation rates, we calculated the proportion of RCCS participants in the census-eligible population by gender, 1-year age band and survey round (Extended Data Fig. 1c-d, bars). Overall, participation rates were lower in men than women (63% vs. 75%). Participation rates also increased with age for both men and women, and were very similar across survey rounds. Considering the grouping patterns by age in the population count data, we again smoothed the participation rates across ages independently for every gender and survey round using LOESS regression as specified above for population size estimation (Extended Data Fig. 1c-d, line).

#### HIV status and prevalence.

All RCCS participants were offered free HIV testing. Prior to October 2011, HIV testing was performed through enzyme immunoassays (EIAs) with confirmation via Western Blot and DNA PCR. After October 2011, testing was performed through a combination of three rapid tests with confirmation of positives, weakly positives and discordant results by at least two EIAs and Western Blot or DNA PCR^76^. Overall, 99.7% participants took up the test offer across survey rounds, and Table S1 documents the number of participants with HIV found. From these survey data, we estimated HIV prevalence (i.e., probability for a participant to have HIV) with a non-parametric Bayesian model over the age of participants independently for both genders and survey round. Specifically, we used a binomial likelihood on the number of participants with HIV parameterised by the number of participants and HIV prevalence in each 1-year age band. The HIV prevalence parameter was modelled on the logit scale by the sum of a baseline and a zero-mean Gaussian Process on the age space. The prior on the baseline was set to a zero-mean normal distribution with a standard deviation of 10. The covariance matrix of the Gaussian Process was defined with a squared exponential kernel, using a zero-mean half-normal with a standard deviation of 2 on the scale parameter of the squared exponential kernel and a zero-mean Half-Normal with a standard deviation of 11.3 ((49 – 15)/3) on the lengthscale of the squared exponential kernel. The model was fitted with Rstan release 2.21.0 using Stan’s adaptive Hamiltonian Monte Carlo (HMC) sampler^77^ with 10,000 iterations, including warm-up 500 iterations. Convergence and mixing were good, with highest R-hat value of 1.0029, and lowest effective sample size of 830). The model represented the data well, with 98.57% of data points inside 95% posterior predictive intervals. Supplementary Fig. S1 shows the age- and gender-specific HIV prevalence estimates in RCSS participants for each survey round. For the mathematical modelling of transmission flows, we assumed that age- and gender-specific HIV prevalence were the same in non-participants in the RCCS communities as in the participants in these communities.

#### ART use.

The RCCS measures ART use through participant reports since survey round 11. Self-reported ART use reflected viral suppression with high specificity and a sensitivity around 70% in the study population (Supplementary Table S7. We took the following pre-processing steps. For survey round 10, we assumed self-reported ART use to have been on the same levels as in round 11. Next, the ART use field was adjusted to “yes” for the participants with HIV who did not report ART use but who had a viral load measurement below 1,000 copies per milliliter (mL) plasma blood. Further, we considered it likely that with increasingly comprehensive care and changing treatment guidelines^20,78^, ART use in individuals with HIV who did not participate increased substantively over time, and this prompted us to consider as proxy of ART use in non-participants the observed ART use in first-time participants with HIV. Overall, first-time participants represented between 15.26% to 39.87% of all participants in rounds 13 and 15 respectively. Extended Data Fig. 8a-b exemplifies the self-reported ART use data in male participants and male first-time participants, along with the combined estimate of individuals with HIV in the study population who report ART use, summing over participants and non-participants. These estimates were obtained using the same Bayesian non-parametric model as for HIV prevalence. Convergence and mixing were good, with highest R-hat value of 1.0025 and lowest effective sample size of 978 for the participants and 1.0027, 521 respectively for first-time participants. The model represented the data well, with 99.67% of data points for the participants inside the corresponding 95% posterior predictive intervals, and 99.24% for the first-time participants. The resulting, estimated ART use rates in infected men and women are shown in Extended Data Fig. 8c.

#### Viral suppression.

Since survey round 15, HIV-1 viral load was measured on stored serum/plasma specimens from infected participants using the Abbott real-time m2000 assay (Abbott Laboratories, IL, USA), which is able to detect a minimum of 40 copies/mL. Viral suppression was defined as a viral load measurement below 1,000 copies/mL plasma blood following recommendations of the World Health Organisation (WHO)^51^. To estimate virus suppression rates in the infected non-participants, we considered again as proxy data on infected first-time participants. Overall, viral load measurements were obtained from 19.3% participants with HIV in survey round 15 and nearly all (>97.71%) participants with HIV since survey round 16^79,80,81^. From these data we estimated the proportion of individuals in the study population with HIV who had suppressed virus, summing over participants and non-participants, using the same Bayesian non-parametric model as for HIV prevalence and ART use. Convergence and mixing were good with lowest R-hat value of 1.0016 and lowest effective sample size of 461 for the participants and 1.0052, 844 respectively for the first-time participants. The model represented the data well, with 98.19% of data points inside 95% posterior predictive intervals and 97.99% for the first-time participants. For the purpose of mathematical modelling of transmission flows, we next considered the earlier survey rounds 10 to 14, for which viral load measurements were not available, and estimated the proportion of the study population with HIV that was virally suppressed by adjusting the estimated ART use data with the sensitivity of being virally suppressed given self-reported ART use and the specificity of being virally suppressed given self-reported no ART use estimated from round 15 where available, and otherwise from round 16 (Table S7). Specificity and sensitivity values by 1-year age bands were linearly interpolated between the midpoints of the age brackets in Table S7. The resulting, estimated virus suppression rates in men and women with HIV are shown in Extended Data Fig. 8d, illustrating that the gap in virus suppression levels increased over time.

#### Sexual behaviour.

RCCS participants reported to interviewers in each round on aspects of sexual behaviour, including the number of sexual partners in the past 12 months within the same community, the number of partners outside the community, and in round 15 also demographic characteristics of up to four partners (Table S8). To interpret HIV transmission flows in the context of typical sexual contact networks, we focused on the detailed behaviour data collected in round 15 and estimated sexual contact intensities between men and women by 1-year age band, defined as the expected number of sexual contacts of one individual of gender g and age a with the population of the opposite gender h and age b in the same community. Estimates were obtained with the Bayesian rate consistency model, version 1.0.0, using default prior specifications^82^. We noted along with previous work^83,84,85,86^ that women tended to report considerably fewer contacts than men (Table S8), prompting us to include in the linear predictor of contact rates additional age-specific random effects to capture under-reporting behaviour in women. Further, community-specific baseline parameters were added to allow for variation in the average level of contact rates in each community, but the age-specific structure of contact rates was assumed to be identical across communities. The resulting model was fitted to all data pertaining to within-community sexual contacts in the last year, including reports of within-community contacts for which information on the partners remained unreported. Contacts reported with partners from outside the same community were excluded, because male-female contacts have to add up to female-male contacts only in the same population denominator, and hence under-reporting could only be adjusted for when within-community contacts are considered. The model was fitted with CmdstanR version 0.5.1^87^ using Stan’s adaptive HMC sampler^77^ with 4 chains, where each chain runs 2800 iterations, including 300 warm-up iterations. Convergence and mixing were good, with highest R-hat value of 1.003, and lowest effective sample size of 1,745. The model represented the data well, with > 99% of data points inside 95% posterior predictive intervals. Table S8 reports the estimated sexual contact intensities from men and women in survey round 15, and shows that the estimated, under-reporting adjusted sexual contact intensities in women were considerably higher than those directly reported. The table also shows that the estimated number of sexual contacts from men to women equal those from women to men, and the estimated age distribution of sexual contacts is shown in Fig. 2 and Extended Data Fig. 7.

### Longitudinal HIV incidence cohort

#### Data and outcomes from the incidence cohort.

The RCCS encompasses both a full census of the study communities and a population-based survey in each surveillance round, which enables identification and follow up of unique individuals over time, and thus provides a comprehensive sampling frame to measure HIV incidence. The RCCS incidence cohort comprises of all RCCS study participants who were HIV-negative at their first visit (baseline) and had at least one subsequent follow-up visit. Individuals in the incidence cohort were considered to be at risk of acquiring HIV after their first visit, and stopped accruing risk at the date of HIV acquisition or the date of last visit. Exposure times were estimated from data collected at survey visit times similarly as in^20^. Individuals in the incidence cohort who remained negative until the last survey round contributed their time between the first and last survey visit to their exposure period. Individuals in the incidence cohort who were found to have acquired HIV must have done so between the visit date of the last round in which they were negative and the visit date of the current round, and the infection date was imputed at random between the two dates. This included incident cases who had no missed visit between the last negative and current visit (type 1) or one missed visit (type 2) as in^20^, but also cases who had more than one missed visit (type 3). Unknown dates were imputed at random 50 times, and individual exposure periods and incident cases were then attributed to each survey round, summed over the cohort, and then averaged over imputations. Table S3 and Extended Data Fig. 2 illustrate the age- and gender-specific exposure times and incidence events in each survey round. In sensitivity analyses, we considered only those individuals in the incidence cohort who resided in one of the 28 inland communities that were continuously surveyed across all rounds 10 to 18, and found similar incidence dynamics with slightly faster declines in incidence rates in younger men, although this difference was not statistically significant.

#### Modelling and analysis.

The primary statistical objective was to estimate longitudinal age-specific HIV incidence rates by 1-year age bands across (discrete) survey rounds, separately for each gender. We used a log-link mixed-effects Poisson regression model, with individual-level exposure times specified as offset on the log scale, common baseline, and further random effects. The random effects comprised a one-dimensional smooth function on the age space, a one-dimensional smooth function on the survey round space, and an interaction term between age and survey round. The functions were specified as one-dimensional Gaussian processes, similar as in the model for estimating HIV prevalence. Alternative specifications, including two-dimensional functions over the participant’s age and survey round, and without interaction terms between age and survey rounds were also tried. Due to the large number of individual observations, models were fitted using maximum-likelihood estimation (MLE) with the R package mgcv version 1.8-38 in the R language^88^, to each of the 50 data sets with imputed exposure times for each gender. Numerical convergence was examined with the gam.check function. Within and between sample uncertainties in parameter estimates, from the variability of the estimation procedure and the data imputation procedure, were incorporated in the age-, gender- and survey round-specific incidence rate estimates by drawing 1,000 replicate incidence rate estimates from the MLE model parameters and associated standard deviation obtained on each of the 50 imputation data sets, and then calculating median estimates and 95% prediction intervals over the 1,000 × 50 Monte Carlo estimates (Fig. 1c). Model fits were evaluated by comparing predicted HIV incidence infections estimates to the empirical data. To assess model fit, incident cases were predicted using the Poisson model parameterised by replicate MLE incidence estimates. Overall, model fit was very good, with 98.80% [98.10-99.49] data points inside the 95% prediction intervals across all 50 imputed data sets as illustrated in Extended Data Fig. 6a. The Akaike information criterion was used to identify the best model for each gender, and the best model was as described above (Table S4).

### Longitudinal viral phylogenetic transmission cohort

#### Data from the transmission cohort.

Within the RCCS, we also performed population-based HIV deep-sequencing spanning a period of more than 6 years, from August 2011 to April 2018. The primary purpose of viral deep sequencing was to reconstruct transmission networks and identify the population-level sources of infections, thus complementing the data collected through the incidence cohort.

The RCCS viral phylogenetic transmission cohort comprises of all participants with HIV for whom at least one HIV deep sequence sample satisfying minimum quality criteria for deep-sequence phylogenetic analysis is available. For survey rounds 15 to 16 (PANGEA-HIV 1), viral sequencing was performed on plasma samples from participants with HIV who had no viral load measurement and self-reported being ART-naïve at the time of the survey, or who had a viral load measurement above 1,000 copies/mL plasma. We used this criterion because viral deep sequencing was not possible within our protocol on samples with virus less than 1,000 copies/mL plasma, and because self-reported ART use was in this population found to be a proxy of virus suppression with reasonable specificity and sensitivity^20,34^. Plasma samples were shipped to University College London Hospital, London, United Kingdom, for automated RNA sample extraction on QIAsymphony SP workstations with the QIAsymphony DSP Virus/ Pathogen Kit (Cat. No. 937036, 937055; Qiagen, Hilden, Germany), followed by one-step reverse transcription polymerase chain reaction (RT-PCR)^89^. Amplification was assessed through gel electrophoresis on a fraction of samples, and samples were shipped to the Wellcome Trust Sanger Institute, Hinxton, United Kingdom for HIV deep-sequencing on Illumina MiSeq and HiSeq platforms in the DNA pipelines core facility. For survey rounds 17 to 18 (PANGEA-HIV 2), viral load measurements were available for all infected participants and viral sequencing was performed on plasma samples of individuals who had not yet been sequenced and who had a viral load measurement above 1,000 copies/mL plasma. Plasma samples were shipped to the Oxford Genomics Centre, Oxford, United Kingdom, for automated RNA sample extraction on QIAsymphony SP workstations with the QIAsymphony DSP Virus/ Pathogen Kit (Cat. No. 937036, 937055; Qiagen, Hilden, Germany), followed by library preparation with the SMARTer Stranded Total RNA-Seq kit v2 - Pico Input Mammalian (Clontech, TaKaRa Bio), size selection on the captured pool to eliminate fragments shorter than 400 nucleotides (nt) with streptavidin-conjugated beads^90^ to enrich the library with fragments desirable for deep-sequence phylogenetic analysis, PCR amplification of the captured fragments, and purification with Agencourt AMPure XP (Beckman Coulter), as described in the veSEQ-HIV protocol^91^. Sequencing was performed on the Illumina NovaSeq 6000 platform at the Oxford Genomics Centre, generating 350 to 600 base pair (bp) paired-end reads. A subset of samples from survey rounds 15 to 16 with low quality read output under the PANGEA-HIV 1 procedure was re-sequenced with the veSEQ-HIV protocol. To enhance the genetic background used in our analyses, additional samples from the spatially neighbouring MRC/UVRI/LSHTM surveillance cohorts and fishing communities were also included. We restricted our analysis to samples from 2,172 individuals that satisfied minimum criteria on read length and depth for phylogeny reconstruction and subsequent inferences. Specifically, deep-sequencing reads were assembled with the shiver sequence assembly software, version 1.5.7^92^. Next, phyloscanner version 1.8.1^39^ was used to merge paired-end reads, and only merged reads of at least 250 bp in length were retained in order to generate 250bp deep-sequence alignments as established in earlier work^34^. Then, for the purpose of deep-sequence phylogenetic analyses, we required that individuals had a depth of ≥ 30 such reads over at least 3 non-overlapping 250bp genomic windows. For brevity, we refer to infected participants with virus sequenced meeting these criteria as “sequenced meeting minimum quality criteria”. Individuals who did not have sequencing output meeting these criteria were excluded from further analysis, and these were primarily individuals sequenced only in PANGEA-HIV 1. Supplementary Table S5 characterises the HIV deep-sequencing outcomes, and Supplementary Table S6 characterises the representativeness of the transmission cohort in participants with HIV.

#### Reconstruction of transmission networks and source-recipient pairs.

The HIV deep-sequencing pipeline provided sequence fragments that capture viral diversity within individuals, which enables phylogenetic inference into the direction of transmission from sequence data alone^34,43,92^. First, potential transmission networks were identified, and in the second step transmission networks were confirmed and the transmission directions in the networks were characterised as possible. In this study, the first step was modified from previous protocols^34^ to ease computational burden, while the second step was as before performed with phyloscanner, using version 1.8.1.

In the first step^93^, to identify potential transmission networks, HIV consensus sequences were generated as the most common nucleotide in the aligned deep-sequence fragments that were derived for each sample. We then calculated similarity scores between all possible combinations of consensus sequences in consecutive 500 bp genomic windows rather than the entire genome to account for the possibility of recombination events and divergent virus in parts of the genome. Similarity score thresholds to identify putative, genetically close pairs were derived from data of long-term sexual partners enrolled in the RCCS cohort similarly as in^34,93^, and then applied to the population-based sample of all possible combinations of successfully sequenced individuals. Overall, 2525 putative, genetically close individuals were identified, and these formed 305 potential transmission networks.

In the second step, we confirmed the potential transmission networks in phylogenetic deep-sequence analyses. We updated the background sequence alignment used in phyloscanner to a new sequence data set that included 113 representatives of all HIV subtypes and circulating recombinant forms and 200 near full-genome sequences from Kenya, Uganda, and Tanzania, obtained from the Los Alamos National Laboratory HIV Sequence Database (http://www.hiv.lanl.gov/). The deep-sequence alignment options were updated to using MAFFT version 7.475 with iterative refinement^94^, and additional iterative re-alignment using consistency scores in case a large proportion of gap-like columns in the first alignment was detected. Deep-sequence phylogeny reconstruction was updated to using IQ-TREE version 2.0.3 with GTR+F+R6 substitution model, resolving the previously documented deep-sequence phylogenetics branch length artefact^29,95^. Confirmatory analyses of the potential transmission networks were updated to using phyloscanner version 1.8.1 with input argument *zeroLengthAdjustment* set to TRUE. From phyloscanner output, we calculated pairwise linkage scores that summarise how frequently viral phylogenetic subgraphs of two individuals were adjacent and phylogenetically close in the deep-sequence phylogenies corresponding to all 250bp genomic windows that contained viral variants from both individuals^34,39^. Similarly we calculated pairwise direction scores that summarise how frequently viral phylogenetic subgraphs of one individual were ancestral to the subgraphs of the other individual in the deep-sequence phylogenies corresponding to all 250bp genomic windows that contained viral variants from both individuals and in which subgraphs had either ancestral or descendant relationships^34,39^. Phylogenetically likely source-recipient pairs with linkage scores ≥ 0.5 and direction scores ≥ 0.5 were extracted, and only the most likely source-recipient pair with highest linkage score was retained if multiple likely sources were identified for a particular recipient. The resulting source-recipient pairs were checked further against sero-history data from both individuals where available. If sero-history data indicated the opposite direction of transmission, the estimated likely direction of transmission was set to that indicated by sero-history data.

#### Infection time estimates.

The shape and depth of an individual’s subgraph in deep-sequence phylogenies also provide information on the time since infection and sequence sampling, and since the sequence sampling date is known thus also on the infection time^96^. We used the phyloTSI random forest estimation routine with default options, which was trained on HIV seroconverter data from the RCCS and other cohorts, and uses as input the output of the phyloscanner software^41^. Individual-level time since infection estimates were associated with wide uncertainty (Extended Data Fig. 5a), and for this reason we refined estimates for the phylogenetically likely recipient in heterosexual source-recipient pairs using the inferred transmission direction, age data, and where available longitudinal sero-history data. Specifically, we refined plausible infection ranges as indicated in the following schema:


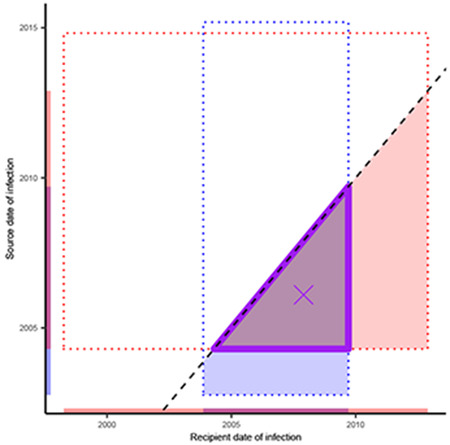


Here, the dotted red rectangle illustrates the 2.5% and 97.5% quantiles of the phyloTSI infection time estimates for the phylogenetically likely recipient (x-axis) and transmitting partner (y-axis). We incorporated evidence on the direction of transmission by requiring that the date of infection of the phylogenetically likely recipient is after that of the transmitting partner (filled red triangle). Sero-history and demographic data were incorporated as follows. For both the recipient and the transmitting partner, the upper bound of the infection date was set as the 30^th^ day prior to the first positive test of the participant^97^. The lower bound of the infection date was set to the largest of the following dates, the date of last negative test if available, the 15^th^ birthday, or the date corresponding to 15 years prior the upper bound^98^. The refined uncertainty range of the infection time estimates of the phylogenetically likely transmitting partner and recipient are illustrated as the purple triangle in the schema above, and obtained as follows. Firstly, we defined individual-level plausible ranges, by intersecting the range of dates consistent with the phyloTSI predictions and sero-history data. If the intersection was empty, we discarded the phyloTSI estimates. Then we intersected the rectangle given by the cartesian product of the plausible intervals for source and recipient with the half-plane consistent with the direction of transmission. Finally, infection dates were sampled at random from the refined uncertainty range, so that the median infection date estimates correspond to the center of gravity of the triangle (cross). In sensitivity analyses, we further integrated estimates of transmission risk by stage of infection^99^, though this had limited impact on the estimates (see Sensitivity Analyses). In cases where the likely transmitting partner in one heterosexual pair was the recipient partner in another heterosexual pair, the above infection date refinement algorithm was applied recursively so that the refined infection date estimates were consistent across pairs. Finally, the transmission events captured by each source-recipient pair were attributed to the survey round into which the posterior median infection time estimate of the recipient fell, and in cases where the median estimate fell after the start time of a round and the end time of the preceding round, the event was attributed to the preceding round.

In total, we identified 539 source-recipient pairs that involved participants from the 36 survey communities and further individuals from the background data set. In 13 of the 539 source-recipient pairs, available dates of last negative tests indicated that only the opposite transmission direction was possible and in these cases the inferred direction of transmission was set to the opposite direction. The resulting pairs included 501 unique recipient partners, and for reach we retained the most likely transmitting partner. To identify pairs capturing transmission events within the RCCS inland communities, we restricted analysis initially to 324 source-recipient pairs in whom both individuals were ever resident in the 36 survey communities. Of these, 55 source-recipient pairs involved two women, 33 pairs involved two men, and 236 pairs were heterosexual, with 142 from men to women and 94 from women to men. Infection times were estimated for all sampled individuals and refined for the recipient partners in the 236 heterosexual source-recipient pairs. For 4 recipient partners, the phyloTSI estimates were ignored as they were incompatible with inferred transmission direction and survey data, and was based on sero-history data only. The phylogenetically most likely location of both individuals at time of transmission was estimated as their location at the RCCS visit date that was closest to the posterior median infection time estimate. Using this location estimate, 233 of the 236 heterosexual source-recipient pairs were estimated to capture transmission events in RCCS inland communities and were retained for further analysis. A further 6 recipient partners had posterior median infection time estimates outside the observation period from September 2003 to May 2018 and were excluded, leaving for analysis 227 heterosexual source-recipient pairs that capture transmission events in RCCS inland communities during the observation period.

## Transmission flow analysis

### Statistical framework.

We next estimated the sources of the inferred population-level HIV incidence dynamics (as described in Section 1) from the dated, source-recipient pairs in the viral phylogenetic transmission cohort. Overall, inference was done in a Bayesian framework using a semi-parametric Poisson flow model similar to Xi, X. *et al.*^45^, that was fitted to observed counts of transmission flows $Y_{p,i,j}^{g\to h}$ with transmission direction g→h (male-to-female or female-to-male), time period *p* (R10-R15 and R16-R18) in which the recipient was likely infected, and 1-year age bands i, j of the source and recipient populations respectively, where

(1a) i,j∈𝒜={15,16,…,48,49}

(1b) (g→h)∈𝒟={male-to-female,female-to-male}.

The target quantity of the model is the expected number of HIV transmissions in the study population in transmission direction g→h (male-to-female or female-to-male), survey round r (R10 to R18) in which infection occurred, and 1-year age bands i, j of the source and recipient populations respectively, which we denote by $\lambda_{r,i,j}^{g\to h}$. We considered that the expected number of HIV transmissions in the study population is characterized by transmission risk and modulated by the number of infectious and susceptible individuals, which prompted us to express $\lambda_{r,i,j}^{g\to h}$ in the form of a standard discrete-time susceptible-infected (SI) model,

(2) $$\lambda_{r,i,j}^{g\to h}=\beta_{r,i,j}^{g\to h}\times S_{r,j}^{h}\times I_{r,i}^{g}\times |(t_{r}^{\text{end}}-t_{r}^{\text{start}})|,$$

where $\beta_{r,i,j}^{g\to h}\;>\;0$ is the transmission rate exerted by one infected, virally unsuppressed individual of gender g and age i on one person in the uninfected (“susceptible”) population of the opposite gender h and age j in a standardized unit of time in round r. With model (2), we express expected transmission flows with a population-level mechanism of how transmission rates from individuals with unsuppressed HIV act on the susceptible population, and we preferred model (2) over a purely phenomenological model of the $\lambda_{r,i,j}^{g\to h}$ for the generalizing insights it provides. The main simplifying approximations in (2) are that all quantities on the right-hand side of (2) are in discrete time and constant in each round, meaning we approximate over changes in population size, HIV prevalence, and viral suppression at a temporally finer scale, and assume further that one generation of transmissions occurs from individuals with unsuppressed HIV in each round. Importantly, in this framework, we can then relate the expected transmission flows to the HIV incidence dynamics and the data from the longitudinal incidence cohort by summing in (2) over the sources of infections,

(3a) $$\sum_{i}\lambda_{r,i,j}^{g\to h}=\left(\sum_{i}\beta_{r,i,j}^{g\to h}\times I_{r,i}^{g}\right)\times S_{r,j}^{h}\times |(t_{r}^{\text{end}}-t_{r}^{\text{start}})|$$

(3b) $$≕\kappa_{r,j}^{h}\times S_{r,j}^{h}\times |(t_{r}^{\text{end}}-t_{r}^{\text{start}})|,$$

where $\kappa_{r,j}^{h}$ is the incidence rate per census-eligible, susceptible person of gender h and age j in round $r\;(S_{r,j}^{h})$ and per unit time $\left(|(t_{r}^{\text{end}}-t_{r}^{\text{start}})|\right)$. Estimates of $\kappa_{r,j}^{h}$ were calculated in units of 100 person-years as described above and shown in Fig. 1c, and we will constrain the semi-parametric Poisson flow model using these estimates. From the model output, we are primarily interested in the transmission flows and transmission sources during each round as quantities out of 100%, defined respectively by

(4a) $$\pi_{r,i,j}^{g\to h}=\lambda_{r,i,j}^{g\to h}∕\left(\sum_{i,j\in 𝒜,(g\to h)\in 𝒟}\lambda_{r,i,j}^{g\to h}\right)$$

(4b) $$\delta_{r,i,j}^{g\to h}=\pi_{r,i,j}^{g\to h}∕\left(\sum_{k\in 𝒜}\pi_{r,k,j}^{g\to h}\right)$$

(4c) $$\delta_{r,i}^{g\to h}=\sum_{j\in 𝒜}\pi_{r,i,j}^{g\to h}.$$

In words, (4b) quantifies the sources of infection in individuals of gender h and age j in round r such that the sum of $\delta_{r,i,j}^{g\to h}$ over i equals one, and (4c) quantifies the sources of infection in the entire population in round r that originate from the group of individuals of gender g and age i such that the sum of $\delta_{r,i}^{g\to h}$ over g and i equals one. The width of the boxplots in Fig. 2b shows (4b) and Fig. 2a, c show (4c).

#### Specification of susceptible and infected individuals.

The number $S_{r,j}^{h}$ of the susceptible population of gender h and age j was calculated by multiplying the smoothed estimate $N_{r,j}^{g}$ of the census-eligible population of gender h and age j (shown in Extended Data Fig. 1a-b) with 1 minus the posterior median estimate of HIV prevalence $\rho_{r,j}^{h}$ in census-eligible individuals of gender h and age j of round r (calculated as described further above and shown in Supplementary Fig. S1). To specify the number $I_{r,i}^{g}$ of individuals with unsuppressed HIV of gender g and age i, we multiplied the smoothed estimate $N_{r,i}^{g}$ of the census-eligible population of gender g and age i of round r (shown in Extended Data Fig. 1a-b) with the posterior median estimate of HIV prevalence in the census-eligible population of gender g and age $i\;(\rho_{r,i}^{g})$ with 1 minus the posterior median estimate $\nu_{r,i}^{g}$ of the proportion of census-eligible individuals of gender g and age i in round r that have suppressed HIV (calculated as described further above and shown in Extended Data Fig. 8d). The start and end times of each survey round, $t_{r}^{\text{start}}$ and $t_{r}^{\text{end}}$ were set as shown in Fig. 1b and specified in units of years, so that the transmission intensity is also expressed in units of years.

#### Bayesian model.

We first present the likelihood of the observed counts of transmission flows $Y_{p,i,j}^{g\to h}$ under the semi-parametric Poisson flow model that is parameterised in terms of (2). The phylogenetically reconstructed source-recipient pairs capture only a subset of incidence events, and so it is important to characterise the sampling frame. Because we are here integrating data from the transmission and incidence cohorts, we are able to adjust inferences by detection probabilities of incidence events. Specifically, we express the detection probability as the ratio of phylogenetically reconstructed transmission events with a recipient of gender h and age j divided by the expected number of incident cases of gender h and age j in time period p as derived in (3),

(5) $$\xi_{p,j}^{h}=\left(\sum_{i\in 𝒜}Y_{p,i,j}^{g\to h}\right)/\left(\sum_{r\in p}\kappa_{r,j}^{h}\times S_{r,j}^{h}\times |(t_{r}^{\text{end}}-t_{r}^{\text{start}})|\right).$$

We assume in (5) that the detection probability does not depend on characteristics of the source, further characteristics of the recipient beyond their age and gender, and is constant in time period p. These assumptions imply that infection events are sampled identically and independently with probability (5), which in turn allows us to express the likelihood of observing the phylogenetic data similarly as in Xi, X. *et al.*^45^ with

(6a) $$Y_{p,i,j}^{g\to h}∼\text{Poisson}\left(\xi_{p,j}^{h}\sum_{r\in p}\lambda_{r,i,j}^{g\to h}\right)$$

(6b) $$\lambda_{r,i,j}^{g\to h}=\beta_{r,i,j}^{g\to h}\times S_{r,j}^{h}\times I_{r,i}^{g}\times |(t_{r}^{\text{end}}-t_{r}^{\text{start}})|$$

(6c) $$\log\;\beta_{r,i,j}^{g\to h}={\hat{c}}^{g\to h}(i,j)+\gamma_{0}+\gamma_{g}+\gamma_{r}+\gamma_{p(r)}+\;\;f_{0}^{g\to h}(i,j)+f_{r}^{g\to h}(j)+f_{p(r)}^{g\to h}(i),$$

where ${\hat{c}}^{g\to h}(i,j)$ is the posterior median estimate of the log rate of sexual contacts within communities in one year between one person of age i and gender g and one person of age j and gender h that we estimated from the sexual behaviour data, and the remaining terms quantify the transmission probability per sexual contact on the log scale. The model is designed in such a way that the log sexual contact rates describe a fixed age-specific non-zero mean surface, and the remaining parameters describe age-specific random deviations around the mean surface. With this approach, any inferred deviations in transmission rates relative to sexual contact rates are informed by the phylogenetic data and robust to prior specifications on the random deviations. Specifically, $\gamma_{0}$ is the baseline parameter characterising overall transmission risk per sexual contact, $\gamma_{g}$ is a gender-specific offset which is set to zero in the female-to-male direction and a real value in male-to-female direction, $\gamma_{r}$ a round-specific offset which is set to zero for the first survey round 10, and $\gamma_{p}$ is a time period specific offset which is set to zero for the first time period. We assume the age-specific structure of transmission rates in terms of the transmitting partners (denoted by i) and recipients (denoted by j) are similar across similar ages, and so we can exploit regularising prior densities^45^ to learn smooth, latent transmission rate surfaces from the sparse data shown in Extended Data Fig. 4. In detail, we modelled the age-specific structure of transmission rates non-parametrically with 2 time-invariant random functions $f_{0}^{g\to h}$ with two-dimensional inputs on the domain [15, 50] × [15, 50] that characterise age-age interactions in transmission risk for each gender, 2 × 8 random functions $f_{r}^{g\to h}$ with one-dimensional inputs that characterise time trends in the age of recipients for each gender for survey rounds after round 10, and 2 random functions $f_{p}^{g\to h}$ with one-dimensional inputs that characterise time trends in the age of transmitting partners for each gender for the second time period. We attach to each of these random functions computationally efficient B-splines projected Gaussian process (GP) priors^100^, which we constructed by describing the random functions with cubic B-splines over equidistant knots and modelling the prior relationship of the B-splines parameters with GPs with squared exponential kernels with variance and lengthscale hyper-parameters, denoted respectively by $\sigma^{2}$ and ℓ. The prior densities of our Bayesian model are

(7a) $$\gamma_{0}∼𝒩(0,10^{2})$$

(7b) $$\gamma_{\text{male}}∼𝒩(0,1)$$

(7c) $$\gamma_{r}∼𝒩(0,1)\;\;\;\;\;\;\;\;\;\;\;\;\;\;\;\;\;\;\;\;\;\;\text{for}\;r>R10$$

(7d) $$\gamma_{p}∼𝒩(0,1)\;\;\;\;\;\;\;\;\;\;\;\;\;\;\;\;\;\;\;\text{for}\;p=R16-R18$$

(7e) $$f_{0}^{g\to h}∼2D-B-\text{splines-GP}(\sigma_{0}^{g\to h},\ell_{0,i}^{g\to h},\ell_{0,j}^{g\to h})$$

(7f) $$f_{r}^{g\to h}∼1D-B-\text{splines-GP}({\tilde{\sigma }}_{r}^{g\to h},{\tilde{\ell }}_{r}^{g\to h}),\;\;\;\;\;\;\;\;\;\;\;\;\text{for}\;r>R10$$

(7g) $$f_{p}^{g\to h}∼1D-B-\text{splines-GP}({\overset{˘}{g}}^{g\to h},{\overset{˘}{\ell }}^{g\to h})\;\;\;\;\;\;\;\;\;\;\;\;\;\;\;\;\text{for}\;p=R16-R18$$

(7h) $$\sigma_{0,i}^{g\to h},\sigma_{0,j}^{g\to h},{\tilde{\sigma }}^{g\to h},{\overset{˘}{\sigma }}^{g\to h}∼\text{Half-Cauchy}(0,1)$$

(7i) $$\ell_{0,i}^{g\to h},\ell_{0,j}^{g\to h},{\tilde{\ell }}^{g\to h},{\overset{˘}{\ell }}^{g\to h}∼\text{Inv-Gamma}(2,2),$$

where the 2 × 8 recipient-specific time-varying 1D B-splines GPs each have squared exponential kernels with hyper-parameters ${\tilde{\sigma }}_{r}^{g\to h}$, ${\tilde{\ell }}^{g\to h}$, the 2 source-specific time-varying 1D B-splines GPs each have squared exponential kernels with hyper-parameters ${\overset{˘}{\sigma }}^{g\to h}$, ${\overset{˘}{\ell }}^{g\to h}$, and the 2 time-invariant 2D B-splines GPs each have squared exponential kernels with hyper-parameters $\sigma_{0,i}^{g\to h}$, $\ell_{0,i}^{g\to h}$ and $\ell_{0,j}^{g\to h}$ decomposed as follows,

(8) $$k_{0}^{g\to h}\left(\left(i,j),(i^{\prime },j^{\prime }\right)\right)=(\sigma_{0}^{g\to h}{)}^{2}\;\text{exp}\left(-\frac{(i-i^{\prime }{)}^{2}}{2(\ell_{0,i}^{g\to h}{)}^{2}}\right)\text{exp}\left(-\frac{(j-j^{\prime }{)}^{2}}{2(\ell_{0,j}^{g\to h}{)}^{2}}\right).$$

We constrain the model further with a pseudo-likelihood term so that the model’s implied incidence rate $\kappa_{r,j}^{h}$ in (3b) is around the MLE incidence rate estimate obtained from the incidence cohort. We took this approach in lieu of fitting the model to both the source-recipient and individual-level incidence exposure data to bypass extreme computational runtimes^18^, and in the context that the source-recipient data are not informative of incidence dynamics^101^. Specifically, we fitted log-normal distributions to the 1,000 × 50 Monte Carlo replicate rate estimates for individuals of gender h and age j in round r (see above) using the lognorm R package version 0.1.6^102^, and then set

(9) $$\frac{\sum_{i}\lambda_{r,i,j}^{g\to h}}{S_{r,j}^{h}\times |(t_{r}^{\text{end}}-t_{r}^{\text{start}})|}∼\text{LogNormal}\left(\text{mean}-{\hat{\kappa }}_{r,j}^{h},\text{var}-{\hat{\kappa }}_{r,j}^{h}\right),$$

where $\text{mean-}{\hat{\kappa }}_{r,j}^{h}$ and $\text{var-}{\hat{\kappa }}_{r,j}^{h}$ denote respectively the parameters of the fitted log-normal distributions, and the left-hand side is calculated from (6b) and matches the model’s incidence rate $\kappa_{r,j}^{h}$ in (3b).

#### Computational inference.

Model (6-9) was fitted with Rstan version 2.21.0, using Stan’s adaptive HMC sampler^77^ with 4 chains for 3,500 iterations including 500 warm-up iterations. Convergence and mixing were good, with highest Rhat value of 1.0041 and lowest effective sample sizes of 1826. The model presented the data well, with 99.57% data point inside 95% posterior predictive intervals. There were no divergent transitions, suggesting non-pathological posterior topologies.

### Counterfactual interventions

We investigated —given the inferred transmission flows— the hypothetical impact of male-targeted counterfactual intervention scenarios c on predicted incidence reductions in women in the most recent survey round 18. In the model, counterfactual interventions were implemented by calculating the expected number of transmission flows (2) into women under counterfactual c that fewer men of age i had remained with unsuppressed HIV in survey round 18, which we denote by ${\tilde{I}}_{R18,i,c}^{M}$. We obtained the expected number of incident cases in women of age j in round 18 in counterfactual c via

(10) $${\tilde{\lambda }}_{R18,j,c}^{M\to F}=\int \sum_{i}{\hat{\beta }}_{R18,i,j}^{M\to F}\times {\tilde{I}}_{R18,i,c}^{M}\times S_{R18,j}^{F}\times |(t_{R18}^{\text{end}}-t_{R18}^{\text{start}})|\;d{\hat{\beta }}_{R18,i,j}^{M\to F},$$

where uncertainty in the posterior age-specific transmission rates after fitting model (6-9) is integrated out. The predicted incidence rate reductions were based on comparing the counterfactuals (10) to the inferred cases in women in the corresponding age group (3b), $1-\left(\sum_{j}{\tilde{\lambda }}_{R18,j,c}^{M\to F}\right)∕\left(\sum_{j}{\hat{\lambda }}_{R18,j}^{M\to F}\right)$.

#### Closing half the gap in viral suppression rates in men relative to women.

In this scenario, we considered the impact of reducing by half the gap in the proportion of men with unsuppressed HIV compared to the same proportion in women. To this end, we first calculated for each 1-year age band the average of the estimated proportion of census-eligible infected men in round 18 with suppressed virus and the same proportion in women, ${\tilde{\nu }}_{R18,i}^{M}=(\nu_{R18,i}^{M}+\nu_{R18,i}^{F})∕2$. Next, we set ${\tilde{I}}_{R18,i,1}^{M}$ to the smoothed estimate of census-eligible men of age i in round R18 multiplied with the posterior median estimate of HIV prevalence in census-eligible men of age i, and with $1-{\tilde{\nu }}_{R18,i}^{M}$.

#### Closing the gap in viral suppression rates in men relative to women.

In this scenario, we considered the impact of achieving the same proportions of men with unsuppressed HIV as in women. To this end, we set ${\tilde{I}}_{R18,i,2}^{M}$ to the smoothed estimate of census-eligible men of age i in round R18 multiplied with the posterior median estimate of HIV prevalence in census-eligible men of age i, and with $1-\nu_{R18,i}^{F}$.

#### 95-95-95 in men.

In this scenario, we considered the impact of achieving viral suppression in 85.7% (0.95 × 0.95 × 0.95) in each 1-year age group of men with HIV. The number of remaining men with unsuppressed HIV in round 18, ${\tilde{I}}_{R18,i,3}^{M}$, was calculated by multiplying the smoothed estimate of the census-eligible men of age i in round R18 with the posterior median estimate of HIV prevalence in the census-eligible men of age i, and with 1 − 0.857.

### Sensitivity analyses

#### Sensitivity in incidence rate estimates to the GAM incidence model specification.

The longitudinal age-specific HIV incidence rates of the central analysis were estimated with a log-link generalised additive effects Poisson regression model with a linear predictor comprising relatively simple main and interaction effects by age and survey round, fitted to individual-level 0/1 incidence outcomes and exposure times specified as offset on the log scale. To assess sensitivity against the relatively simple linear predictor, we considered a more complex mean specification comprising independent LOESS smoothers to capture age-specific incidence trends in each survey round, and fitted this mean model for computational reasons to crude HIV incidence rates. Specifically, we fitted LOESS regressions as implemented in the R package stats version 3.6.2 with span argument set to 0.7 to the crude age-, gender- and round-specific HIV incidence rates in all 50 imputation data sets, and weighted by the corresponding, group-level aggregated exposure times. The HIV incidence rate estimates under the LOESS model had as expected a smaller mean absolute error against the crude estimates as compared against the GAM model (0.0048 [0.0046-0.0051] versus 0.0053 [0.0051-0.0056]) (Supplementary Fig. S2). Overall, the contribution of men to incidence was more variable across rounds while the shifts in the median age at infection were similar in the central and this sensitivity analysis (Supplementary Table S11).

#### Sensitivity in incidence rate and transmission flow estimates to limited communities.

Over time some communities were added and others left the RCCS (see Supplementary Table S2). We repeated our analysis on the subset of 28 consecutively surveyed communities. We found similar incidence rates with slightly faster declines in male new infections and larger gender disparities, although the prediction intervals around the estimated incidence rates in the sensitivity analysis largely overlapped with those in the central analysis (Supplementary Figure S3). All other primary findings remained insensitive (Supplementary Table S11).

#### Sensitivity in estimating transmission flows to uncertainty in infection time estimates.

In the central analysis, phyloTSI infection time estimates associated to source-recipient pairs were refined using the inferred transmission direction, age, and sero-history data. To assess sensitivity to the infection time estimates used, we inferred transmission flows on the basis of the raw phyloTSI infection time estimates as long as they were compatible with the inferred transmission direction, and otherwise on the basis of the refined estimates. Overall, we found source-recipient pairs were potentially allocated to earlier or later time periods reflecting the wide uncertainty in infection time estimates, though across the sample the age distribution of sources and recipients was remarkably stable (Extended Data Fig. 5). All primary findings were insensitive to using the raw infection time estimates (Supplementary Table S11).

#### Sensitivity in time since infection estimates to higher transmissibility during acute infection.

In the central analysis, transmission flows were estimated using the centre of gravity of the uncertainty region associated with the refined infection time estimates. To account for higher transmission rates during acute infection of the transmitting partner^99^, we assumed that the transmission hazard was 5 times higher in the first 2 months after infection of the transmitting partner as compared to the following period, and obtained the resulting mean infection time estimate under this assumption by generalising our Monte Carlo approach used in the central analysis to an importance sampling approach under piecewise linear transmission hazards. The primary results were insensitive to these changes as less than 5% of source-recipent pairs attributed to different survey rounds(Supplementary Table S11).

#### Sensitivity in estimating transmission flows to right censoring of likely transmission pairs.

The RCCS transmission cohort was defined retrospectively and so it is possible that some transmission events, especially in later rounds, remain as of yet unseen because the corresponding individuals are not yet in the survey or do not yet have virus deep-sequenced. To assess sensitivity to right censoring, we excluded from analysis those source-recipient pairs for which virus of the source or the recipient was deep-sequenced only after rounds 17, 16 and 15. The primary findings were insensitive to these analyses because the probabilities of detecting infection event in the phylogenetic data changed accordingly (Supplementary Table S11 and Supplementary Figure S4).

#### Sensitivity in estimated transmission flows to limited sample size of likely transmission pairs.

The number of observed infection events in the incidence cohort was ≈4 times larger than the number of reconstructed transmission events, prompting us to explore the effect of sampling uncertainty on the transmission flow estimates. We bootstrap sampled source-recipient pairs at random with replacement three times, and repeated inferences on these bootstrap samples. Our primary findings remained insensitive (Supplementary Table S11).

#### Sensitivity in transmission flow estimates to the phylo-SI model specification.

In the central analysis, the log transmission rates that underpin the estimated transmission flows were estimated using the linear predictor in (6c), and this model specification was associated with overall smallest mean absolute error and posterior predictive coverage as shown in Supplementary Table S10 against the following alternative models,

(11a) $$\log\;\beta_{r,i,j}^{g\to h}={\hat{c}}^{g\to h}(i,j)+\gamma_{0}+\gamma_{g}+\gamma_{r}+\gamma_{p(r)}+\;\;f_{0}^{g\to h}(i,j)+f_{p(r)}^{g\to h}(i),$$

(11b) $$\log\;\beta_{r,i,j}^{g\to h}={\hat{c}}^{g\to h}(i,j)+\gamma_{0}+\gamma_{g}+\gamma_{r}+\gamma_{p(r)}+\;\;f_{0}^{g\to h}(i,j)+f_{p(r)}^{g\to h}(j),$$

(11c) $$\log\;\beta_{r,i,j}^{g\to h}={\hat{c}}^{g\to h}(i,j)+\gamma_{0}+\gamma_{g}+\gamma_{r}+\gamma_{p(r)}+\;\;f_{0}^{g\to h}(i,j)+f_{p(r)}^{g\to h}(i,j),$$

(11d) $$\log\;\beta_{r,i,j}^{g\to h}={\hat{c}}^{g\to h}(i,j)+\gamma_{0}+\gamma_{g}+\gamma_{r}+\gamma_{p(r)}+\;\;f_{0}^{g\to h}(i,j)+f_{r}^{g\to h}(j)$$

(11e) $$\log\;\beta_{r,i,j}^{g\to h}={\hat{c}}^{g\to h}(i,j)+\gamma_{0}+\gamma_{g}+\gamma_{r}+\gamma_{p(r)}+\;\;f_{0}^{g\to h}(i,j)+f_{r}^{g\to h}(j)+f_{p(r)}^{g\to h}(j),$$

(11f) $$\log\;\beta_{r,i,j}^{g\to h}={\hat{c}}^{g\to h}(i,j)+\gamma_{0}+\gamma_{g}+\gamma_{r}+\gamma_{p(r)}+\;\;f_{0}^{g\to h}(i,j)+f_{r}^{g\to h}(j)+f_{p(r)}^{g\to h}(i,j).$$

Models specifying transmission rates without a round-specific random function on the age of infected individuals, (11a)-(11c), did not fit the data well (Supplementary Table S10). The remaining models, (11d)-(11f) performed as well as the model used in the central analysis (Supplementary Table S10) and our primary findings remained insensitive (Supplementary Table S11).

#### Sensitivity in counterfactual intervention impacts to assumptions on viral suppression rates in non-participants.

Infection and suppression levels in the non-participant census-eligible population remained unknown and in the central analysis, we considered as proxy of virus suppression rates among non-participants data from first-time participants. We performed two sensitivity analyses, assuming first that virus suppression rates were zero in non-participants across all rounds, and assuming second that virus suppression rates were identical in non-participants and participants of the same age, gender and survey round. Together, the two scenarios likely encompass the true, unknown viral suppression rates in non-participants. These scenarios were implemented by updating the number of virally unsuppressed individuals in (2), and refitting the model. The sensitivity analysis assuming all non-participants with HIV had unsuppressed virus resulted in larger predicted incidence reductions in women around 70%, while the sensitivity analysis assuming all non-participants with HIV had the same suppression rates as participants with HIV resulted in similar predicted incidence reductions in women than in the central analysis (Supplementary Table S11).

#### Sensitivity in counterfactual intervention impacts to potentially higher HIV prevalence in non-participants.

In the central analysis, we assumed that HIV prevalence was the same in participants and non-participants of the same age, gender and survey round. We considered three sensitivity analyses, assuming first that prevalence was 25% higher in male non-participants compared to male participants of the same age, gender and survey round, assuming second that prevalence was 25% higher in female non-participants compared to female participants of the same age, gender and survey round, and assuming third that prevalence was 25% higher in female and male non-participants compared to female and male participants of the same age, gender and survey round respectively. These scenarios were implemented by updating the number of virally unsuppressed individuals in (2), and refitting the model. Our primary findings remained insensitive (Supplementary Table S11).

#### Sensitivity in counterfactual intervention impacts to lower viral suppression rate thresholds.

Different definitions of HIV suppression are currently operational, and we considered the effect of lower thresholds to define viral suppression (<200 copies/mL) than in the central analysis (<1,000 copies/mL). The detection limit of our viral load measurement instruments pre-empted considering a threshold of <50 copies/mL. This scenario was implemented by re-estimating the age- and gender-specific proportions of individuals with HIV in the study population who had suppressed virus at the lower threshold, re-calculating gaps in viral suppression rates in men relative to women, and re-calculating the additional number of men needed to reach and maintain virally suppressed in the counterfactual intervention scenarios. We found slightly smaller gender gaps in viral suppression at the lower threshold and the predicted incidence reduction in women in the counterfactual that assessed closing the suppression gap in men was around 45%, and all other findings remained insensitive (Supplementary Table S11).

## Extended Data

## Supplementary Material

1

## Figures and Tables

**Table 1:** Policy summary.

Background	Little data exist on how the drivers of HIV infection are changing in Africa, and how multi-billion dollar HIV services and intervention programs can respond to maximise equitable and effective control of HIV transmission.
Main findings and limitations	We quantitatively reconstructed the drivers of HIV transmission by age and gender using population-based HIV deep-sequence phylogenetics in Rakai, Uganda, and interpreted the phylogenetic data in the context of prospective HIV incidence surveillance and additional data on population-level virus suppression and sexual behaviour. We document evolving transmission dynamics by age and gender between 2003 and 2018. First, along with increasing availability of HIV services, there have been consistently faster increases in viral suppression among women than men, and by 2018 viral suppression rates were 1.5-2 fold higher among women than men. Second, in parallel HIV incidence rates declined significantly faster among men than women with an increasing majority of new infections arising from men, thus widening pre-existing gender disparity in HIV transmission. Third, the contribution of transmission flows to adolescent girls and young women by older partners declined by one third. Fourth, the contribution of transmission flows to women aged 25-34 years from partners aged 0-6 years older doubled. Fifth, in simulations we estimate that closing the suppression gap in men relative to women could have reduced infections in women by one half and ended gender disparity in HIV incidence. Limitations include incomplete participation and viral sequencing of the study population, and that this longitudinal surveillance and deep-sequence data are from a single cohort.
Policy implications	In Africa, too many men with HIV are left behind and do not reach viral suppression. Based on the reconstructed transmission flows, our findings suggest that had there been effective efforts to reach and maintain men in HIV services with viral suppression levels corresponding to those of their female counterparts, half of new female infections could have been averted while also closing the growing gender disparities in incidence. More male-centred HIV services are thus likely a central component to prevent infections and end HIV and AIDS as a public health threat by 2030.

**Table 2:** HIV prevalence, viral suppression, transmission sources, and impact of counterfactual male-targeted interventions by age of male partner, round 18, Oct 2016-Apr 2018.

						Closing half the suppression gap		Closing the suppression gap		95-95-95 in men	
Age	HIV prevalence in men	Men with HIV who have unsuppressed virus	Male-female difference in proportion of individuals with HIV who have unsuppressed virus	Contribution of age group to all men with unsuppressed virus	Contribution of age group to all transmitting Male partners	Contribution of age group to additional number of men with unsuppressed virus in counterfactual	Predicted reduction in incidence in women in round 18	Contribution of age group to additional number of men with unsuppressed virus in counterfactual	Predicted reduction in incidence in women in round 18	Contribution of age group to additional number of men with unsuppressed virus in counterfactual	Predicted reduction in incidence in women in round 18
	(% in age bracket)	(% in age bracket)	(difference)	(%)	(%)	(%)	(% of actual incidence)	(%)	(% of actual incidence)	(%)	(% of actual incidence)
15-19	0.8 [0.4-1.3]	73.2 [56.6-86.2]	36.8 [16.8-54.2]	5.3 [3.0-8.8]	1.3 [0.4-3.4]	5.3 [2.5-8.7]	21.8 [21.1-22.7]	5.3 [2.5-8.7]	43.5 [42.2-45.3]	7.4 [5.2-9.9]	72.6 [69.2-74.9]
20-24	2.1 [1.6-2.7]	66.5 [55.5-76.6]	26.6 [14.0-38.2]	9.0 [6.5-11.8]	13.1 [8.1-19.6]	7.1 [3.7-10.9]	24.3 [22.9-25.7]	7.1 [3.7-10.9]	48.8 [45.9-51.8]	12.2 [9.4-15.6]	61.7 [57.0-66.3]
25-29	6.2 [5.2-7.3]	53.5 [45.3-61.4]	23.2 [13.7-32.6]	17.9 [14.6-21.5]	18.9 [13.5-25.1]	15.0 [9.1-21.5]	25.1 [23.8-26.4]	15.0 [9.1-21.5]	50.4 [47.9-53.1]	22.7 [18.2-28.1]	58.5 [54.5-62.5]
30-34	12.5 [10.8-14.2]	39.6 [33.6-45.8]	19.2 [12.1-26.1]	24.3 [20.8-28.0]	20.4 [15.4-26.8]	21.3 [13.7-27.7]	25.8 [24.4-27.2]	21.3 [13.7-27.7]	52.0 [49.1-54.9]	26.9 [22.2-31.5]	56.4 [51.9-61.2]
35-39	16.4 [14.6-18.2]	28.9 [23.3-35.0]	17.6 [11.2-24.3]	21.3 [17.9-24.8]	26.2 [19.4-34.1]	24.0 [16.9-31.1]	26.8 [25.0-28.6]	24.0 [16.9-31.1]	54.1 [50.3-57.7]	18.6 [13.5-23.6]	53.4 [47.6-59.5]
40-44	16.8 [14.8-18.9]	21.9 [16.3-28.2]	14.6 [8.5-21.5]	13.8 [10.6-17.1]	14.9 [9.5-21.3]	17.7 [10.9-23.9]	28.2 [25.8-29.9]	17.7 [10.9-23.9]	57.5 [52.3-60.9]	8.3 [2.6-13.4]	43.3 [36.2-52.6]
45-49	16.4 [14.1-19.1]	19.4 [13.0-27.0]	12.2 [4.5-20.5]	8.2 [5.5-11.3]	4 [1.3-8]	9.6 [2.7-15.8]	27.3 [24.7-29.1]	9.6 [2.7-15.8]	56.2 [50.2-59.8]	3.7 [0.0-8.5]	40.8 [29.2-55.8]
Total	8.0 [7.4-8.6]	33.9 [29.7-38.3]	14.8 [10.0-19.6]	100	100	100	25.2 [24.2-26.2]	100	50.6 [48.7-52.7]	100	58.4 [55.1-61.6]

## Data Availability

The deep-sequence phylogenies and basic individual-level data analysed during the current study are available at https://github.com/MLGlobalHealth/phyloSI-RakaiAgeGender. HIV-1 reads are available on reasonable request through the PANGEA-HIV consortium. Please contact project manager Lucie Abeler-Dörner (lucie.abeler-dorner@bdi.ox.ac.uk) for further details. Additional individual-level data are available on reasonable request to RHSP.

## Code availability

Code to reproduce all analyses is freely available on GitHub version 1.1.2 under the GNU General Public License version 3.0 at the repository https://github.com/MLGlobalHealth/phyloSI-RakaiAgeGender.
