# Supplementary material for: Growing gender disparity in HIV infection in Africa: sources and policy implications
Source: medRxiv. 2023 Mar 20:2023.03.16.23287351. Preprint. [Version 2] doi: 10.1101/2023.03.16.23287351 (PMC10055554; doi:10.1101/2023.03.16.23287351)
Supplement: 1 [file NIHPP2023.03.16.23287351v2-supplement-1.pdf]

medRxiv preprint doi: https://doi.org/10.1101/2023.03.16.23287351; this version posted March 20, 2023. The copyright holder for this preprint (which was not certified by peer review) is the author/funder, who has granted medRxiv a license to display the preprint in perpetuity.
It is made available under a CC-BY 4.0 International license .

4 Supplementary Tables

medRxiv preprint doi: https://doi.org/10.1101/2023.03.16.23287351; this version posted March 20, 2023. The copyright holder for this preprint (which was not certified by peer review) is the author/funder, who has granted medRxiv a license to display the preprint in perpetuity.
It is made available under a CC-BY 4.0 International license .

	Census-eligible individuals	Participants	Participants with HIV	Participants with HIV and with measured viral load	Participants with HIV reporting to be ART naïve	Participants with HIV and with unsuppressed virus†	Participants with HIV and with virus ever deep-sequenced‡
**Round 10, September 26, 2003 - November 23, 2004; 28 communities surveyed**							
Total	11,976	7,407	884	–	–	–	115
Female	6,299	4,341	575	–	–	–	60
Age							
15-24	3,118	1,768	131	–	–	–	17
25-34	1,916	1,538	280	–	–	–	27
35-49	1,265	1,035	164	–	–	–	16
Male	5,677	3,066	309	–	–	–	55
Age							
15-24	2,672	1,186	38	–	–	–	9
25-34	1,845	1,132	145	–	–	–	27
35-49	1,160	748	126	–	–	–	19
**Round 11, February 15, 2005 - June 30, 2006; 28 communities surveyed**							
Total	12,528	8,273	1,002	–	884	–	176
Female	6,644	4,786	658	–	568	–	97
Age							
15-24	3,146	1,818	141	–	138	–	26
25-34	2,175	1,842	323	–	286	–	50
35-49	1,323	1,126	194	–	144	–	21
Male	5,884	3,487	344	–	316	–	79
Age							
15-24	2,670	1,293	30	–	30	–	6
25-34	1,956	1,290	160	–	153	–	40
35-49	1,258	904	154	–	133	–	33
**Round 12, August 30, 2006 - June 06, 2008; 28 communities surveyed**							
Total	13,718	8,752	1,105	–	912	–	234
Female	7,185	5,047	746	–	610	–	140
Age							
15-24	3,331	1,903	151	–	149	–	37
25-34	2,416	1,958	354	–	297	–	67
35-49	1,438	1,186	241	–	164	–	36
Male	6,533	3,705	359	–	302	–	94
Age							
15-24	2,866	1,426	26	–	25	–	8
25-34	2,200	1,305	168	–	156	–	50
35-49	1,467	974	165	–	121	–	36
**Round 13, June 17, 2008 - July 12, 2009; 28 communities surveyed**							
Total	13,433	8,718	1,160	–	900	–	368
Female	7,086	4,975	760	–	580	–	203
Age							
15-24	3,160	1,736	128	–	124	–	45
25-34	2,379	1,946	347	–	278	–	99
35-49	1,547	1,293	285	–	178	–	59
Male	6,347	3,743	400	–	320	–	165
Age							
15-24	2,749	1,397	32	–	31	–	19
25-34	2,042	1,275	177	–	160	–	82
35-49	1,556	1,071	191	–	129	–	64
**Round 14, January 18, 2010 - June 21, 2011; 28 communities surveyed**							
Total	14,828	10,091	1,741	–	1392	–	820
Female	7,766	5,716	1,155	–	901	–	478
Age							
15-24	3,376	1,919	176	–	167	–	95
25-34	2,633	2,221	516	–	427	–	238
35-49	1,757	1,576	463	–	307	–	145
Male	7,062	4,375	586	–	491	–	342
Age							
15-24	2,963	1,629	52	–	50	–	37
25-34	2,276	1,455	242	–	220	–	152
35-49	1,823	1,291	292	–	221	–	153

† Unsuppressed virus was defined as a plasma viral load measurement above 1000 copies/mL plasma blood. ‡ Individuals with virus ever deep-sequenced were defined as HIV-positive individuals with deep-sequence output meeting minimum quality criteria, see Methods.

Supplementary Table S1: Characteristics of the RCCS study population.

medRxiv preprint doi: https://doi.org/10.1101/2023.03.16.23287351; this version posted March 20, 2023. The copyright holder for this preprint (which was not certified by peer review) is the author/funder, who has granted medRxiv a license to display the preprint in perpetuity.
It is made available under a CC-BY 4.0 International license .

	Census-eligible individuals	Participants	Participants with HIV	Participants with HIV and with measured viral load	Participants with HIV reporting to be ART naïve	Participants with HIV and with unsuppressed virus†	Participants with HIV and with virus ever deep-sequenced‡
**Round 15, August 10, 2011 - July 05, 2013; 33 communities surveyed**							
Total	20,806	13,589	1,944	367	1331	207	1,085
Female	10,782	7,538	1,287	232	844	122	637
Age							
15-24	4,751	2,742	217	31	186	23	157
25-34	3,631	2,825	568	101	405	64	307
35-49	2,400	1,971	502	100	253	35	173
Male	10,024	6,051	657	135	487	85	448
Age							
15-24	4,150	2,368	68	11	58	10	54
25-34	3,243	1,955	260	57	218	41	207
35-49	2,631	1,728	329	67	211	34	187
**Round 16, July 08, 2013 - January 30, 2015; 35 communities surveyed**							
Total	21,887	14,072	1,875	1829	868	671	892
Female	11,346	7,816	1,255	1224	537	390	521
Age							
15-24	5,089	2,891	194	189	129	97	83
25-34	3,547	2,669	502	486	238	175	249
35-49	2,710	2,256	559	549	170	118	189
Male	10,541	6,256	620	605	331	281	371
Age							
15-24	4,436	2,462	50	47	40	34	35
25-34	3,241	1,883	219	212	141	123	154
35-49	2,864	1,911	351	346	150	124	182
**Round 17, February 23, 2015 - September 02, 2016; 35 communities surveyed**							
Total	22,929	15,093	2,015	2004	646	514	933
Female	11,990	8,377	1,390	1384	408	304	553
Age							
15-24	5,393	3,035	205	204	94	84	97
25-34	3,544	2,723	529	525	194	147	250
35-49	3,053	2,619	656	655	120	73	206
Male	10,939	6,716	625	620	238	210	380
Age							
15-24	4,677	2,662	41	40	28	26	31
25-34	3,121	1,912	208	206	102	91	139
35-49	3,141	2,142	376	374	108	93	210
**Round 18, October 03, 2016 - May 22, 2018; 35 communities surveyed**							
Total	23,269	15,053	1,860	1850	432	375	848
Female	12,193	8,331	1,275	1271	263	206	491
Age							
15-24	5,484	3,049	158	158	72	63	80
25-34	3,472	2,592	461	457	117	95	207
35-49	3,237	2,690	656	656	74	48	204
Male	11,076	6,722	585	579	169	169	357
Age							
15-24	4,739	2,671	38	36	22	24	27
25-34	3,077	1,850	183	183	79	78	128
35-49	3,260	2,201	364	360	68	67	202

† Unsuppressed virus was defined as a plasma viral load measurement above 1000 copies/mL plasma blood. ‡ Individuals with virus ever deep-sequenced were defined as HIV-positive individuals with deep-sequence output meeting minimum quality criteria, see Methods.

Supplementary Table S1: **Characteristics of the RCCS study population (continued).**

medRxiv preprint doi: https://doi.org/10.1101/2023.03.16.23287351; this version posted March 20, 2023. The copyright holder for this preprint (which was not certified by peer review) is the author/funder, who has granted medRxiv a license to display the preprint in perpetuity.
It is made available under a CC-BY 4.0 International license .

Community Identifier†	Part of RCCS								
	Round 10	Round 11	Round 12	Round 13	Round 14	Round 15	Round 16	Round 17	Round 18
i-01	Yes	Yes	Yes	Yes	Yes	Yes	Yes	Yes	Yes
i-02	Yes	Yes	Yes	Yes	Yes	Yes	Yes	Yes	Yes
i-03	Yes	Yes	Yes	Yes	Yes	Yes	Yes	Yes	Yes
i-04	Yes	Yes	Yes	Yes	Yes	Yes	Yes	Yes	Yes
i-05	Yes	Yes	Yes	Yes	Yes	Yes	Yes	Yes	Yes
i-06	Yes	Yes	Yes	Yes	Yes	Yes	Yes	Yes	Yes
i-07	Yes	Yes	Yes	Yes	Yes	Yes	Yes	Yes	Yes
i-08	Yes	Yes	Yes	Yes	Yes	Yes	Yes	Yes	Yes
i-09	No	No	No	No	No	Yes	Yes	Yes	Yes
i-10	Yes	Yes	Yes	Yes	Yes	Yes	Yes	Yes	Yes
i-11	Yes	Yes	Yes	Yes	Yes	Yes	Yes	Yes	Yes
i-12	Yes	Yes	Yes	Yes	Yes	Yes	Yes	Yes	Yes
i-13	No	No	No	No	No	No	Yes	Yes	Yes
i-14	No	No	No	No	No	Yes	Yes	Yes	Yes
i-15	Yes	Yes	Yes	Yes	Yes	Yes	Yes	Yes	Yes
i-16	Yes	Yes	Yes	Yes	Yes	Yes	Yes	Yes	Yes
i-17	Yes	Yes	Yes	Yes	Yes	Yes	Yes	Yes	Yes
i-18	No	No	No	No	No	No	Yes	Yes	Yes
i-19	Yes	Yes	Yes	Yes	Yes	Yes	Yes	Yes	Yes
i-20	No	No	No	No	No	Yes	No	No	No
i-21	No	No	No	No	No	No	Yes	Yes	Yes
i-22	Yes	Yes	Yes	Yes	Yes	Yes	Yes	Yes	Yes
i-23	Yes	Yes	Yes	Yes	Yes	Yes	Yes	Yes	Yes
i-24	Yes	Yes	Yes	Yes	Yes	Yes	Yes	Yes	Yes
i-25	Yes	Yes	Yes	Yes	Yes	Yes	Yes	Yes	Yes
i-26	Yes	Yes	Yes	Yes	Yes	Yes	Yes	Yes	Yes
i-27	Yes	Yes	Yes	Yes	Yes	Yes	Yes	Yes	Yes
i-28	Yes	Yes	Yes	Yes	Yes	Yes	Yes	Yes	Yes
i-29	Yes	Yes	Yes	Yes	Yes	Yes	Yes	Yes	Yes
i-30	Yes	Yes	Yes	Yes	Yes	Yes	Yes	Yes	Yes
i-31	Yes	Yes	Yes	Yes	Yes	Yes	Yes	Yes	Yes
i-32	No	No	No	No	No	Yes	Yes	Yes	Yes
i-33	No	No	No	No	No	Yes	Yes	Yes	Yes
i-34	Yes	Yes	Yes	Yes	Yes	Yes	Yes	Yes	Yes
i-35	Yes	Yes	Yes	Yes	Yes	Yes	Yes	Yes	Yes
i-36	Yes	Yes	Yes	Yes	Yes	Yes	Yes	Yes	Yes

† Three pairs of geographically close areas in peri-urban settings were merged into three communities.

Supplementary Table S2: Communities surveyed by RCCS in rounds 10-18.

medRxiv preprint doi: https://doi.org/10.1101/2023.03.16.23287351; this version posted March 20, 2023. The copyright holder for this preprint (which was not certified by peer review) is the author/funder, who has granted medRxiv a license to display the preprint in perpetuity.
It is made available under a CC-BY 4.0 International license .

	Incidence cohort†	Person-years‡	Incidence events§	Incidence rate estimate per 100 PY¶
**Round 10, September 26, 2003 - November 23, 2004; 28 communities surveyed**				
Total	3,153	9,464.33 [9,448.40-9,481.02]	122.0 [112.22-130.77]	1.32 [1.27-1.37]
Female	1,760	5,213.53 [5,201.59-5,224.77]	71.0 [61.22-77.00]	1.37 [1.30-1.45]
Age				
15-24	772	1,938.12 [1,928.82-1,944.71]	32.0 [28.22-37.77]	1.53 [1.40-1.68]
25-34	551	2,025.25 [2,015.02-2,032.45]	26.0 [21.00-34.77]	1.50 [1.38-1.63]
35-49	437	1,251.31 [1,247.69-1,255.27]	11.0 [8.00-13.77]	0.90 [0.81-1.01]
Male	1,393	4,252.08 [4,237.69-4,264.78]	51.0 [45.22-57.32]	1.26 [1.19-1.33]
Age				
15-24	610	1,522.78 [1,514.43-1,527.77]	12.0 [7.22-14.77]	1.04 [0.94-1.15]
25-34	547	1,718.06 [1,708.34-1,725.71]	29.0 [24.23-34.77]	1.61 [1.48-1.75]
35-49	236	1,011.40 [1,006.45-1,015.40]	10.0 [8.00-13.00]	1.00 [0.89-1.11]
**Round 11, February 15, 2005 - June 30, 2006; 28 communities surveyed**				
Total	4,359	11,484.46 [11,465.55-11,505.89]	144.0 [131.45-154.77]	1.29 [1.23-1.34]
Female	2,390	6,261.90 [6,247.27-6,278.78]	84.0 [76.22-91.00]	1.36 [1.27-1.44]
Age				
15-24	1,079	2,088.35 [2,078.11-2,095.10]	31.0 [25.45-37.00]	1.48 [1.34-1.64]
25-34	754	2,654.38 [2,644.12-2,664.53]	39.0 [34.00-43.77]	1.46 [1.34-1.59]
35-49	557	1,519.83 [1,515.14-1,526.38]	13.0 [11.00-17.00]	1.00 [0.90-1.11]
Male	1,969	5,222.93 [5,209.14-5,243.01]	60.0 [51.45-65.00]	1.20 [1.14-1.27]
Age				
15-24	896	1,781.07 [1,774.24-1,787.04]	17.0 [12.00-20.00]	0.97 [0.88-1.06]
25-34	734	2,145.73 [2,135.78-2,156.34]	31.0 [26.23-36.77]	1.55 [1.43-1.69]
35-49	339	1,296.59 [1,291.00-1,302.85]	11.0 [8.00-14.77]	0.95 [0.86-1.06]
**Round 12, August 30, 2006 - June 06, 2008; 28 communities surveyed**				
Total	5,492	12,396.23 [12,369.28-12,422.54]	168.0 [151.12-177.33]	1.21 [1.16-1.28]
Female	2,984	6,648.49 [6,632.13-6,668.15]	95.0 [84.67-101.00]	1.31 [1.24-1.43]
Age				
15-24	1,310	2,100.25 [2,091.86-2,108.29]	31.0 [25.00-36.77]	1.44 [1.29-1.62]
25-34	1,007	2,883.98 [2,869.79-2,897.39]	45.0 [39.23-52.77]	1.39 [1.27-1.54]
35-49	667	1,666.57 [1,659.44-1,673.22]	19.0 [16.00-22.00]	1.02 [0.90-1.18]
Male	2,508	5,746.59 [5,732.78-5,759.56]	72.0 [65.22-79.00]	1.09 [1.02-1.17]
Age				
15-24	1,111	1,990.10 [1,984.61-1,996.47]	15.0 [10.22-17.00]	0.83 [0.75-0.92]
25-34	933	2,246.22 [2,235.56-2,252.48]	38.0 [32.00-44.00]	1.46 [1.34-1.59]
35-49	464	1,511.44 [1,503.26-1,516.12]	19.0 [16.23-23.77]	0.88 [0.80-1.00]
**Round 13, June 17, 2008 - July 12, 2009; 28 communities surveyed**				
Total	6,544	11,823.39 [11,802.83-11,845.07]	136.0 [125.00-145.55]	1.08 [1.04-1.15]
Female	3,579	6,331.90 [6,313.25-6,348.15]	83.0 [73.45-89.00]	1.21 [1.15-1.33]
Age				
15-24	1,531	1,942.24 [1,932.52-1,949.30]	29.0 [25.00-35.77]	1.38 [1.26-1.54]
25-34	1,246	2,723.50 [2,708.26-2,732.27]	37.0 [32.00-43.55]	1.27 [1.17-1.41]
35-49	802	1,667.46 [1,661.12-1,673.50]	16.0 [12.00-21.77]	0.90 [0.81-1.06]
Male	2,965	5,490.33 [5,477.08-5,500.21]	52.0 [47.23-59.55]	0.94 [0.89-1.02]
Age				
15-24	1,249	1,900.09 [1,893.07-1,905.26]	17.0 [13.23-21.55]	0.69 [0.63-0.77]
25-34	1,119	2,004.64 [1,996.54-2,012.04]	23.0 [18.00-27.77]	1.30 [1.20-1.43]
35-49	597	1,586.25 [1,578.36-1,593.72]	13.0 [10.00-16.77]	0.78 [0.70-0.89]
**Round 14, January 18, 2010 - June 21, 2011; 28 communities surveyed**				
Total	7,471	12,359.17 [12,344.41-12,374.39]	107.5 [97.45-118.00]	0.93 [0.89-0.97]
Female	4,109	6,624.63 [6,608.63-6,638.01]	63.0 [55.00-71.78]	1.07 [1.00-1.13]
Age				
15-24	1,722	1,998.64 [1,991.11-2,007.00]	23.0 [19.00-30.00]	1.30 [1.17-1.43]
25-34	1,481	2,766.97 [2,761.12-2,775.18]	24.0 [15.68-30.55]	1.13 [1.03-1.22]
35-49	906	1,857.69 [1,850.32-1,863.26]	15.0 [11.00-19.00]	0.74 [0.66-0.82]
Male	3,362	5,734.81 [5,725.76-5,744.10]	46.0 [39.23-50.00]	0.77 [0.73-0.82]
Age				
15-24	1,337	1,988.89 [1,983.58-1,992.83]	14.0 [10.00-16.77]	0.55 [0.50-0.61]
25-34	1,276	1,999.30 [1,992.87-2,005.68]	22.0 [19.00-26.77]	1.11 [1.02-1.21]
35-49	749	1,747.16 [1,742.21-1,752.55]	9.0 [6.22-12.77]	0.65 [0.58-0.73]

† Number of RCCS study participants who were HIV-negative at their first visit and had at least one subsequent follow-up visit. ‡ Number of person-years of HIV acquisition risk. § Number of incidence events. The infection date was imputed at random to have occurred between the last negative and first positive survey visit dates, and the incidence event was attributed to the corresponding survey round 50 times. The range of the person-years and incidence events across the 50 data sets with imputed exposure times are presented. ¶ Estimated incidence rate per 100 person-years. The confidence interval of the estimated incidence rate incorporates both the variability of the estimation procedure and the data imputation procedure.

Supplementary Table S3: Characteristics of the longitudinal HIV incidence cohort.

medRxiv preprint doi: https://doi.org/10.1101/2023.03.16.23287351; this version posted March 20, 2023. The copyright holder for this preprint (which was not certified by peer review) is the author/funder, who has granted medRxiv a license to display the preprint in perpetuity.
It is made available under a CC-BY 4.0 International license .

	Incidence cohort†	Person-years‡	Incidence events§	Incidence rate estimate per 100 PY¶
**Round 15, August 10, 2011 - July 05, 2013; 33 communities surveyed**				
Total	7,961	17,621.81 [17,596.06-17,643.04]	140.0 [129.45-148.78]	0.79 [0.76-0.83]
Female	4,393	9,227.87 [9,204.36-9,242.47]	87.0 [79.22-94.77]	0.94 [0.88-0.99]
Age				
15-24	1,656	2,742.21 [2,728.96-2,752.62]	37.0 [31.23-43.77]	1.17 [1.05-1.30]
25-34	1,737	3,728.50 [3,713.89-3,735.50]	38.0 [34.00-42.77]	1.02 [0.92-1.10]
35-49	1,000	2,757.15 [2,750.51-2,765.25]	12.0 [9.23-15.77]	0.61 [0.54-0.68]
Male	3,568	8,395.89 [8,383.12-8,406.96]	52.0 [47.23-60.00]	0.64 [0.60-0.67]
Age				
15-24	1,328	2,842.07 [2,836.70-2,847.92]	11.0 [8.00-14.00]	0.45 [0.41-0.50]
25-34	1,404	2,865.12 [2,856.30-2,874.43]	31.0 [26.23-35.00]	0.92 [0.84-1.01]
35-49	836	2,687.98 [2,679.81-2,695.84]	11.0 [6.22-14.00]	0.52 [0.46-0.59]
**Round 16, July 08, 2013 - January 30, 2015; 35 communities surveyed**				
Total	8,709	16,633.57 [16,621.16-16,648.28]	108.5 [98.45-116.78]	0.66 [0.63-0.70]
Female	4,729	8,745.06 [8,737.02-8,758.26]	72.5 [64.22-80.78]	0.80 [0.75-0.86]
Age				
15-24	1,679	2,699.66 [2,693.50-2,703.90]	24.5 [21.23-31.55]	0.89 [0.80-0.99]
25-34	1,950	3,202.15 [3,195.15-3,209.33]	33.0 [27.00-38.77]	0.94 [0.85-1.04]
35-49	1,100	2,843.90 [2,839.95-2,847.65]	15.0 [11.22-18.00]	0.55 [0.49-0.62]
Male	3,980	7,888.21 [7,881.14-7,895.54]	35.0 [31.00-39.00]	0.51 [0.48-0.55]
Age				
15-24	1,462	2,803.63 [2,801.36-2,806.94]	8.0 [7.00-10.00]	0.37 [0.32-0.41]
25-34	1,533	2,501.71 [2,496.99-2,507.33]	17.0 [13.00-20.00]	0.77 [0.68-0.84]
35-49	985	2,582.08 [2,578.79-2,588.18]	9.0 [6.22-14.00]	0.43 [0.37-0.49]
**Round 17, February 23, 2015 - September 02, 2016; 35 communities surveyed**				
Total	9,077	17,437.70 [17,422.40-17,448.35]	89.5 [80.22-95.78]	0.56 [0.53-0.59]
Female	4,918	9,116.75 [9,106.85-9,127.51]	57.0 [48.45-61.77]	0.68 [0.64-0.72]
Age				
15-24	1,634	2,796.00 [2,790.86-2,799.37]	11.0 [8.00-13.77]	0.62 [0.56-0.70]
25-34	2,066	3,187.45 [3,182.16-3,194.41]	28.0 [23.23-32.00]	0.87 [0.80-0.95]
35-49	1,218	3,133.05 [3,127.18-3,138.08]	17.0 [15.00-21.77]	0.53 [0.48-0.59]
Male	4,159	8,321.01 [8,312.47-8,328.62]	32.0 [27.45-36.00]	0.43 [0.40-0.46]
Age				
15-24	1,493	3,012.95 [3,009.30-3,015.97]	9.0 [8.00-11.00]	0.30 [0.27-0.35]
25-34	1,550	2,485.06 [2,479.65-2,490.25]	14.0 [10.22-18.00]	0.65 [0.58-0.73]
35-49	1,116	2,823.11 [2,818.49-2,830.23]	9.0 [5.22-12.00]	0.36 [0.30-0.42]
**Round 18, October 03, 2016 - May 22, 2018; 35 communities surveyed**				
Total	9,665	17,992.52 [17,982.46-18,005.50]	89.0 [83.00-97.78]	0.50 [0.47-0.54]
Female	5,186	9,624.65 [9,617.33-9,633.49]	57.0 [53.00-65.00]	0.62 [0.56-0.68]
Age				
15-24	1,704	2,703.74 [2,699.61-2,706.79]	12.0 [10.00-13.77]	0.42 [0.35-0.51]
25-34	2,135	3,249.56 [3,241.74-3,255.03]	26.0 [24.00-30.77]	0.85 [0.75-0.96]
35-49	1,347	3,671.67 [3,665.44-3,676.22]	19.0 [16.23-23.00]	0.56 [0.47-0.65]
Male	4,479	8,368.03 [8,361.41-8,377.69]	32.0 [30.00-35.00]	0.37 [0.34-0.40]
Age				
15-24	1,633	2,895.16 [2,891.38-2,899.31]	10.0 [8.00-12.00]	0.26 [0.22-0.31]
25-34	1,577	2,496.56 [2,493.55-2,501.84]	14.0 [12.00-17.00]	0.56 [0.49-0.64]
35-49	1,269	2,976.37 [2,972.27-2,980.31]	8.0 [6.00-11.00]	0.31 [0.25-0.37]

† Number of RCCS study participants who were HIV-negative at their first visit and had at least one subsequent follow-up visit. ‡ Number of person-years of HIV acquisition risk. § Number of incidence events. The infection date was imputed at random to have occurred between the last negative and first positive survey visit dates, and the incidence event was attributed to the corresponding survey round 50 times. The range of the person-years and incidence events across the 50 data sets with imputed exposure times are presented. ¶ Estimated incidence rate per 100 person-years. The confidence interval of the estimated incidence rate incorporates both the variability of the estimation procedure and the data imputation procedure.

Supplementary Table S3: Characteristics of the longitudinal HIV incidence cohort (continued).

medRxiv preprint doi: https://doi.org/10.1101/2023.03.16.23287351; this version posted March 20, 2023. The copyright holder for this preprint (which was not certified by peer review) is the author/funder, who has granted medRxiv a license to display the preprint in perpetuity.
It is made available under a CC-BY 4.0 International license .

	Akaike information criterion (AIC)		% observations within 95% prediction intervals		
	Men	Women	Men	Women	All
Central model					
	**8,032** [7,937-8,140]	**11,579** [11,508-11,688]	98.77% [97.78-99.68]	**98.82%** [97.78-99.68]	**98.80%** [98.10-99.49]
Alternative models					
with 2D GP over age and survey round	8,033 [7,938-8,141]	11,580 [11,511-11,690]	**98.84%** [98.10-99.68]	93.32% [91.18-95.10]	96.08% [94.96-96.95]
without interaction term between age and survey round	8,033 [7,938-8,142]	11,592 [11,521-11,706]	98.79% [97.78-99.68]	93.83% [92.06-95.24]	96.31% [95.27-97.23]
with 2D GP over age and survey round and without interaction term between age and survey round	8,035 [7,939-8,143]	11,590 [11,517-11,701]	98.82% [97.78-99.68]	93.45% [90.94-95.24]	96.13% [94.99-97.23]

Supplementary Table S4: **Model comparison for estimating longitudinal, age-specific incidence rates.**

medRxiv preprint doi: https://doi.org/10.1101/2023.03.16.23287351; this version posted March 20, 2023. The copyright holder for this preprint (which was not certified by peer review) is the author/funder, who has granted medRxiv a license to display the preprint in perpetuity. It is made available under a CC-BY 4.0 International license .

	Participants with HIV (n)	Participants with HIV reporting no ART use (n)	Participants with HIV and with virus ever deep-sequenced with Illumina MiSeq in PANGEA-HIV 1 † (n)	Participants with HIV and with virus ever deep-sequenced with Illumina HiSeq in PANGEA-HIV 1 ‡ (n)	Participants with HIV and with virus ever deep-sequenced with Illumina NovaSeq in PANGEA-HIV 2 § (n)	Participants with HIV and with virus ever deep-sequenced (n)
**Round 10, September 26, 2003 - November 23, 2004; 28 communities surveyed**						
Total	884	884	78	6	86	121
Female	575	575	45	4	55	64
Age						
15-24	131	131	15	2	14	19
25-34	280	280	17	1	32	28
35-49	164	164	13	1	9	17
Male	309	309	33	2	31	57
Age						
15-24	38	38	7	0	4	9
25-34	145	145	14	2	17	28
35-49	126	126	12	0	10	20
**Round 11, February 15, 2005 - June 30, 2006; 28 communities surveyed**						
Total	1002	884	115	4	126	180
Female	658	568	69	2	80	98
Age						
15-24	141	138	11	1	22	26
25-34	323	286	42	0	46	50
35-49	194	144	16	1	12	22
Male	344	316	46	2	46	82
Age						
15-24	30	30	5	0	2	7
25-34	160	153	24	2	22	41
35-49	154	133	17	0	22	34
**Round 12, August 30, 2006 - June 06, 2008; 28 communities surveyed**						
Total	1105	912	172	4	157	238
Female	1002	884	115	4	126	180
Age						
15-24	746	610	103	2	107	142
25-34	151	149	20	1	25	37
35-49	354	297	61	0	53	68
241	164	22	1	29	37	
Male	359	302	69	2	50	96
Age						
15-24	26	25	8	0	2	8
25-34	168	156	36	2	25	51
35-49	165	121	25	0	23	37
**Round 13, June 17, 2008 - July 12, 2009; 28 communities surveyed**						
Total	1160	900	247	3	237	371
Female	760	580	145	2	146	206
Age						
15-24	128	124	30	1	24	45
25-34	347	278	76	0	75	101
35-49	285	178	39	1	47	60
Male	400	320	102	1	91	165
Age						
15-24	32	31	16	0	6	19
25-34	177	160	51	1	45	82
35-49	191	129	35	0	40	64
**Round 14, January 18, 2010 - June 21, 2011; 28 communities surveyed**						
Total	1313	964	453	3	361	603
Female	869	615	284	2	225	342
Age						
15-24	134	125	72	0	36	71
25-34	379	290	137	1	113	168
35-49	356	200	75	1	76	103
Male	444	349	169	1	136	261
Age						
15-24	40	38	24	0	11	31
25-34	185	163	75	1	66	120
35-49	219	148	70	0	59	110

† RNA samples were sequenced using the protocol of[89] at the Wellcome Trust Sanger Institute, Hinxton, UK on Illumina MiSeq platforms. Deep-sequences reported satisfied minimum quality criteria for deep-sequence phylogenetic analysis, see Methods. ‡ As for previous column, on Illumina HiSeq platforms. § RNA samples were sequenced using the protocol of[91] at the Oxford Genomics Centre, Oxford, UK on Illumina NovaSeq 6000 platforms. Deep-sequences reported satisfied minimum quality criteria for deep-sequence phylogenetic analysis, see Methods.

Supplementary Table S5: Longitudinal HIV deep-sequencing.

medRxiv preprint doi: https://doi.org/10.1101/2023.03.16.23287351; this version posted March 20, 2023. The copyright holder for this preprint (which was not certified by peer review) is the author/funder, who has granted medRxiv a license to display the preprint in perpetuity.
It is made available under a CC-BY 4.0 International license .
LaTeX template

	Participants with HIV (n)	Participants with HIV reporting no ART use (n)	Participants with HIV and with virus ever deep-sequenced with Illumina MiSeq in PANGEA-HIV 1 † (n)	Participants with HIV and with virus ever deep-sequenced with Illumina HiSeq in PANGEA-HIV 1 ‡ (n)	Participants with HIV and with virus ever deep-sequenced with Illumina NovaSeq in PANGEA-HIV 2 § (n)	Participants with HIV and with virus ever deep-sequenced (n)
**Round 15, August 10, 2012 - July 05, 2013; 33 communities surveyed**						
Total	1901	1298	343	2	968	1054
Female	1264	827	203	1	616	621
Age						
15-24	209	178	34	0	139	150
25-34	557	398	106	1	285	300
35-49	498	251	63	0	192	171
Male	637	471	140	1	352	433
Age						
15-24	67	57	21	0	37	53
25-34	249	208	58	0	162	198
35-49	321	206	61	1	153	182
**Round 16, July 08, 2013 - January 30, 2015; 35 communities surveyed**						
Total	1872	1348	507	3	668	894
Female	1253	862	300	1	426	522
Age						
15-24	194	152	64	0	70	83
25-34	501	367	139	1	187	250
35-49	558	343	97	0	169	189
Male	619	486	207	2	242	372
Age						
15-24	49	43	22	0	18	35
25-34	219	185	93	0	91	155
35-49	351	258	92	2	133	182
**Round 17, February 23, 2015 - September 02, 2016; 35 communities surveyed**						
Total	2014	637	722	8	430	937
Female	1390	401	436	5	284	557
Age						
15-24	205	91	97	0	18	97
25-34	529	190	195	3	113	252
35-49	656	120	144	2	153	208
Male	624	236	286	3	146	380
Age						
15-24	40	27	29	0	3	31
25-34	208	102	114	0	46	139
35-49	376	107	143	3	97	210
**Round 18, October 03, 2016 - May 22, 2018; 35 communities surveyed**						
Total	1860	416	653	3	379	852
Female	1275	255	378	2	250	494
Age						
15-24	158	71	73	0	9	80
25-34	461	111	160	1	96	210
35-49	656	73	145	1	145	204
Male	585	161	275	1	129	358
Age						
15-24	38	22	26	0	1	27
25-34	183	76	111	0	32	129
35-49	364	63	138	1	96	202

† RNA samples were sequenced using the protocol of[89] at the Wellcome Trust Sanger Institute, Hinxton, UK on Illumina MiSeq platforms. Deep-sequences reported satisfied minimum quality criteria for deep-sequence phylogenetic analysis, see Methods. ‡ As for previous column, on Illumina HiSeq platforms. § RNA samples were sequenced using the protocol of[91] at the Oxford Genomics Centre, Oxford, UK on Illumina NovaSeq 6000 platforms. Deep-sequences reported satisfied minimum quality criteria for deep-sequence phylogenetic analysis, see Methods.

Supplementary Table S5: Longitudinal HIV deep-sequencing (continued).

medRxiv preprint doi: https://doi.org/10.1101/2023.03.16.23287351; this version posted March 20, 2023. The copyright holder for this preprint (which was not certified by peer review) is the author/funder, who has granted medRxiv a license to display the preprint in perpetuity.
It is made available under a CC-BY 4.0 International license .

	**Participants with HIV** (n)	**Participants with HIV reporting no ART use at first visit** (n)	(n)	**Participants with HIV and with virus ever deep-sequenced †** (%)
Total	5682	4341	2174	38 %
Female (Total)	3817	2836	1291	34 %
Age				
15-24	1066	817	424	40 %
25-34	2074	1488	740	36 %
35-49	1446	826	411	28 %
Male (Total)	1865	1506	883	47 %
Age				
15-24	272	220	157	58 %
25-34	955	782	499	52 %
35-49	984	670	436	44 %
Round‡				
10	884	–	115	13 %
11	1002	884	176	18 %
12	1105	912	234	21 %
13	1160	900	368	32 %
14	1741	1392	820	47 %
15	1944	1331	1085	56 %
16	1875	868	892	48 %
17	2015	646	933	46 %
18	1860	432	848	46 %

† Individuals with virus ever deep-sequenced were defined as HIV-positive individuals with deep-sequence output meeting minimum quality criteria, see Methods. ‡ Totals by round include individuals seen in other rounds.

Supplementary Table S6: Characteristics of the RCCS transmission cohort, survey rounds 10-18, September 26, 2003 - May 22, 2018.

medRxiv preprint doi: https://doi.org/10.1101/2023.03.16.23287351; this version posted March 20, 2023. The copyright holder for this preprint (which was not certified by peer review) is the author/funder, who has granted medRxiv a license to display the preprint in perpetuity.
It is made available under a CC-BY 4.0 International license .

	Participants reporting no ART use and who have suppressed virus	Participants reporting no ART use and who have unsuppressed virus	Participants reporting ART use and who have suppressed virus	Participants reporting ART use and who have unsuppressed virus	Sensitivity	Specificity
**Round 15, August 10, 2011 - July 05, 2013; 33 communities surveyed**						
Total	65	202	95	5	95.0% [88.5- 98.1]	75.7% [70.2- 80.4]
Female	44	118	66	4	94.3% [85.8- 98.2]	72.8% [65.5- 79.1]
Age						
15-24	5	22	3	1	75.0% [28.9- 96.6]	81.5% [62.8- 92.3]
25-34	19	63	18	1	94.7% [73.5-100.0]	76.8% [66.5- 84.7]
35-49	20	33	45	2	95.7% [85.0- 99.6]	62.3% [48.8- 74.1]
Male	21	84	29	1	96.7% [81.9-100.0]	80.0% [71.3- 86.6]
Age						
15-24	1	10	0	0		90.9% [60.1-100.0]
25-34	8	41	8	0	100.0% [62.8-100.0]	83.7% [70.7- 91.8]
35-49	12	33	21	1	95.5% [76.5-100.0]	73.3% [58.8- 84.2]
**Round 16, July 08, 2013 - January 30, 2015; 35 communities surveyed**						
Total	235	596	923	75	92.5% [90.7- 94.0]	71.7% [68.6- 74.7]
Female	171	342	663	48	93.2% [91.1- 94.9]	66.7% [62.5- 70.6]
Age						
15-24	37	87	55	10	84.6% [73.7- 91.6]	70.2% [61.6- 77.5]
25-34	72	152	239	23	91.2% [87.1- 94.1]	67.9% [61.5- 73.6]
35-49	62	103	369	15	96.1% [93.6- 97.7]	62.4% [54.8- 69.5]
Male	64	254	260	27	90.6% [86.6- 93.5]	79.9% [75.1- 83.9]
Age						
15-24	5	32	8	2	80.0% [47.9- 95.4]	86.5% [71.5- 94.6]
25-34	19	115	70	8	89.7% [80.8- 94.9]	85.8% [78.8- 90.8]
35-49	40	107	182	17	91.5% [86.7- 94.7]	72.8% [65.1- 79.4]
**Round 17, February 23, 2015 - September 02, 2016; 35 communities surveyed**						
Total	221	421	1269	93	93.2% [91.7- 94.4]	65.6% [61.8- 69.2]
Female	165	241	915	63	93.6% [91.8- 94.9]	59.4% [54.5- 64.0]
Age						
15-24	28	66	92	18	83.6% [75.5- 89.5]	70.2% [60.3- 78.5]
25-34	73	119	305	28	91.6% [88.1- 94.2]	62.0% [54.9- 68.6]
35-49	64	56	518	17	96.8% [94.9- 98.0]	46.7% [38.0- 55.6]
Male	56	180	354	30	92.2% [89.0- 94.5]	76.3% [70.4- 81.3]
Age						
15-24	3	24	11	2	84.6% [56.5- 96.9]	88.9% [71.1- 97.0]
25-34	19	82	96	9	91.4% [84.3- 95.6]	81.2% [72.4- 87.7]
35-49	34	74	247	19	92.9% [89.1- 95.4]	68.5% [59.2- 76.5]
**Round 18, October 03, 2016 - May 22, 2018; 35 communities surveyed**						
Total	141	288	1334	87	93.9% [92.5- 95.0]	67.1% [62.6- 71.4]
Female	109	153	956	53	94.7% [93.2- 96.0]	58.4% [52.3- 64.2]
Age						
15-24	20	52	75	11	87.2% [78.4- 92.9]	72.2% [60.9- 81.3]
25-34	48	68	314	27	92.1% [88.7- 94.5]	58.6% [49.5- 67.2]
35-49	41	33	567	15	97.4% [95.8- 98.5]	44.6% [33.8- 55.9]
Male	32	135	378	34	91.7% [88.7- 94.1]	80.8% [74.2- 86.1]
Age						
15-24	1	20	11	4	73.3% [47.6- 89.5]	95.2% [75.6-100.0]
25-34	15	64	90	14	86.5% [78.5- 91.9]	81.0% [70.9- 88.3]
35-49	16	51	277	16	94.5% [91.3- 96.7]	76.1% [64.6- 84.8]

Supplementary Table S7: **Self-reported ART use and viral suppression in RCCS participants with HIV.**

medRxiv preprint doi: https://doi.org/10.1101/2023.03.16.23287351; this version posted March 20, 2023. The copyright holder for this preprint (which was not certified by peer review) is the author/funder, who has granted medRxiv a license to display the preprint in perpetuity. It is made available under a CC-BY 4.0 International license .

	**Participants** (n)	**Contacts with reported partner characteristics** (%)	**Reported contacts per participant** (n)	**Estimated contacts per person** (median, 95% CrI)	**Estimated reporting bias** (median, 95% CrI)	**Reported contacts scaled to population** (n)	**Estimated contacts scaled to population** (median, 95% CrI)
Total	13,277	85.1	0.74	0.84 [0.76, 0.95]	0.1 [0.02, 0.21]	16,025	18,183 [16,450, 20,613]
Female	7,375	87.69	0.64	0.81 [0.74, 0.91]	0.17 [0.10, 0.27]	7,189	9,092 [8,284, 10,238]
Age							
15-19	1,296	84.20	0.34	0.48 [0.44, 0.54]	0.14 [0.09, 0.20]	844	1,187 [1,067, 1,321]
20-24	1,378	91.06	0.84	1.17 [1.09, 1.25]	0.33 [0.25, 0.41]	1,787	2,487 [2,324, 2,662]
25-29	1,432	85.99	0.90	1.18 [1.10, 1.26]	0.27 [0.20, 0.36]	1,704	2,221 [2,074, 2,381]
30-34	1,323	87.64	0.84	0.99 [0.92, 1.08]	0.15 [0.07, 0.24]	1,334	1,569 [1,451, 1,705]
35-39	1,007	87.60	0.75	0.83 [0.75, 0.95]	0.08 [0.00, 0.20]	849	942 [847, 1,075]
40-44	562	90.03	0.60	0.65 [0.55, 0.81]	0.05 [-0.05, 0.21]	436	472 [398, 588]
45-49	377	83.73	0.49	0.34 [0.21, 0.61]	-0.15 [-0.28, 0.12]	236	164 [102, 293]
50-54	0	-	-	0.13 [0.06, 0.36]	- -	-	43 [20, 124]
55-59	0	-	-	0.01 [0.00, 0.18]	- -	-	4 [1, 45]
60-64	0	-	-	0.01 [0.00, 0.14]	- -	-	1 [0, 24]
65-69	0	-	-	0.01 [0.00, 0.17]	- -	-	1 [0, 20]
Male	5,902	82.58	0.85	0.88 [0.79, 1.00]	0.02 [-0.06, 0.15]	8,836	9,091 [8,166, 10,374]
Age							
15-19	1,295	66.42	0.20	0.17 [0.14, 0.20]	-0.04 [-0.06, -0.01]	444	363 [306, 431]
20-24	1,001	75.50	0.84	0.79 [0.72, 0.87]	-0.04 [-0.11, 0.03]	1,528	1,447 [1,321, 1,585]
25-29	1,001	82.29	1.17	1.15 [1.07, 1.24]	-0.02 [-0.10, 0.07]	1,928	1,902 [1,763, 2,049]
30-34	913	84.05	1.26	1.28 [1.19, 1.37]	0.02 [-0.08, 0.11]	1,858	1,881 [1,747, 2,022]
35-39	796	83.82	1.36	1.31 [1.21, 1.41]	-0.05 [-0.14, 0.05]	1,587	1,530 [1,418, 1,648]
40-44	554	88.94	1.20	1.22 [1.11, 1.33]	0.01 [-0.09, 0.12]	990	999 [913, 1,089]
45-49	342	91.35	0.97	1.12 [0.98, 1.27]	0.15 [0.01, 0.30]	502	580 [509, 656]
50-54	0	-	-	0.79 [0.47, 1.31]	- -	-	251 [151, 417]
55-59	0	-	-	0.48 [0.15, 1.43]	- -	-	98 [30, 290]
60-64	0	-	-	0.26 [0.06, 1.14]	- -	-	33 [7, 142]
65-69	0	-	-	0.10 [0.02, 0.62]	- -	-	7 [1, 45]

Supplementary Table S8: Sexual behaviour characteristics in RCCS participants, round 15, October 08 2011 - July 05 2013.

medRxiv preprint doi: https://doi.org/10.1101/2023.03.16.23287351; this version posted March 20, 2023. The copyright holder for this preprint (which was not certified by peer review) is the author/funder, who has granted medRxiv a license to display the preprint in perpetuity.
It is made available under a CC-BY 4.0 International license .

Transmission direction	Male-female difference in age at transmission	Infected partner by age at transmission 15-24 years (%)†	25-34 years (%)†	35-49 years (%)†	Total (%)†
**Round 10, September 26, 2003 - November 23, 2004; 28 communities surveyed**					
Male to female	Total	31.9% [30.2-33.6]	18.6% [17.7-19.6]	7.3% [6.7-7.9]	57.9% [56.1-59.6]
	<0 years	0.4% [0.2-0.7]	4.5% [3.0-6.3]	4.1% [2.7-5.5]	9.0% [6.8-11.5]
	0-6 years	16.0% [12.8-19.3]	8.5% [7.0-10.2]	3.0% [1.8-4.2]	27.5% [23.5-31.5]
	>6 years	15.5% [12.2-18.8]	5.6% [4.1-7.3]	0.2% [0.0-0.5]	21.3% [17.2-25.2]
Female to male	Total	14.8% [13.9-15.8]	20.7% [19.7-21.7]	6.6% [6.2-7.1]	42.1% [40.4-43.9]
	<0 years	6.8% [5.2-8.7]	4.3% [2.9-6.0]	0.4% [0.2-0.8]	11.6% [8.8-14.8]
	0-6 years	7.9% [5.9-9.9]	12.3% [10.5-13.9]	2.5% [1.7-3.3]	22.7% [19.7-25.7]
	>6 years	0.1% [0.0-0.2]	4.0% [2.7-5.8]	3.7% [2.7-4.7]	7.8% [5.8-10.1]
Total		46.7% [45.3-48.2]	39.3% [38.2-40.5]	13.9% [13.2-14.7]	100%
**Round 15, August 10, 2011 - July 05, 2013; 33 communities surveyed**					
Male to female	Total	32.2% [30.1-34.3]	22.0% [20.7-23.4]	7.7% [7.0-8.5]	61.9% [60.2-63.7]
	<0 years	0.5% [0.3-1.0]	4.8% [3.2-6.9]	3.9% [2.4-5.5]	9.3% [6.8-12.2]
	0-6 years	16.0% [12.7-19.4]	10.0% [8.1-12.0]	3.5% [2.1-4.9]	29.6% [25.3-33.9]
	>6 years	15.6% [12.2-19.1]	7.1% [5.3-9.1]	0.2% [0.1-0.7]	23.1% [18.6-27.3]
Female to male	Total	11.5% [10.6-12.4]	18.8% [17.8-19.9]	7.7% [7.1-8.4]	38.1% [36.3-39.8]
	<0 years	6.4% [4.9-7.8]	4.2% [2.9-5.9]	0.6% [0.2-1.2]	11.2% [8.7-14.0]
	0-6 years	5.1% [3.8-6.5]	11.8% [10.1-13.2]	3.2% [2.2-4.2]	20.0% [17.3-22.7]
	>6 years	0.0% [0.0-0.0]	2.8% [1.9-3.9]	3.9% [2.8-5.1]	6.8% [5.1-8.6]
Total		43.7% [41.9-45.6]	40.8% [39.3-42.4]	15.4% [14.6-16.4]	100%
**Round 18, October 03, 2016 - May 22, 2018; 35 communities surveyed**					
Male to female	Total	20.6% [18.2-23.4]	27.3% [25.3-29.4]	14.7% [13.3-16.3]	62.8% [60.2-65.2]
	<0 years	0.3% [0.1-0.7]	5.3% [3.2-8.4]	7.2% [4.8-9.7]	12.9% [9.2-17.4]
	0-6 years	8.7% [6.2-11.7]	13.2% [10.5-16.0]	7.0% [4.8-9.3]	29.0% [25.0-33.2]
	>6 years	11.5% [8.6-14.7]	8.6% [6.0-11.7]	0.5% [0.1-1.4]	20.7% [16.1-25.5]
Female to male	Total	11.2% [9.9-12.6]	17.3% [15.8-18.9]	8.7% [7.7-9.9]	37.2% [34.8-39.8]
	<0 years	5.8% [4.2-7.7]	3.5% [2.4-5.2]	0.4% [0.2-1.1]	9.8% [7.3-13.0]
	0-6 years	5.4% [3.6-7.1]	11.0% [9.3-12.6]	3.2% [2.1-4.5]	19.6% [16.7-22.4]
	>6 years	0.0% [0.0-0.1]	2.8% [1.8-4.0]	5.0% [3.5-6.4]	7.8% [5.8-9.9]
Total		31.9% [29.4-34.5]	44.7% [42.5-46.8]	23.4% [21.7-25.3]	100%

† Posterior median flow estimates and 95% credible intervals in each survey round.

Supplementary Table S9: **Longitudinal HIV transmission flows by age and gender.**

medRxiv preprint doi: https://doi.org/10.1101/2023.03.16.23287351; this version posted March 20, 2023. The copyright holder for this preprint (which was not certified by peer review) is the author/funder, who has granted medRxiv a license to display the preprint in perpetuity.
It is made available under a CC-BY 4.0 International license .

Observed transmission events within 95% prediction interval (%)	Observed transmission events vs. predicted transmission events (MAE)†	Incidence rate prior mean within 95% posterior range (%)	Incidence rate prior mean vs. incidence rate posterior median (MAE)†
**Central model**			
$\log \beta_{r,i,j}^{g\to h} = \hat{\boldsymbol{c}}^{g\to h}(i,j) + \gamma_0 + \gamma_g + \gamma_r + \gamma_{p(r)} + \boldsymbol{f}_0^{g\to h}(i,j) + \boldsymbol{f}_r^{g\to h}(j) + \boldsymbol{f}_{p(r)}^{g\to h}(i)$, (6c)			
99.57	0.0482	96.98	0.00032
**Alternative models**			
$\log \beta_{r,i,j}^{g\to h} = \hat{\boldsymbol{c}}^{g\to h}(i,j) + \gamma_0 + \gamma_g + \gamma_r + \gamma_{p(r)} + \boldsymbol{f}_0^{g\to h}(i,j) + \boldsymbol{f}_{p(r)}^{g\to h}(i)$, (11a)			
99.55	0.0495	67.78	0.00058
$\log \beta_{r,i,j}^{g\to h} = \hat{\boldsymbol{c}}^{g\to h}(i,j) + \gamma_0 + \gamma_g + \gamma_r + \gamma_{p(r)} + \boldsymbol{f}_0^{g\to h}(i,j) + \boldsymbol{f}_{p(r)}^{g\to h}(j)$, (11b)			
99.59	0.0486	66.67	0.00058
$\log \beta_{r,i,j}^{g\to h} = \hat{\boldsymbol{c}}^{g\to h}(i,j) + \gamma_0 + \gamma_g + \gamma_r + \gamma_{p(r)} + \boldsymbol{f}_0^{g\to h}(i,j) + \boldsymbol{f}_{p(r)}^{g\to h}(i,j)$, (11c)			
99.57	0.0492	68.1	0.00057
$\log \beta_{r,i,j}^{g\to h} = \hat{\boldsymbol{c}}^{g\to h}(i,j) + \gamma_0 + \gamma_g + \gamma_r + \gamma_{p(r)} + \boldsymbol{f}_0^{g\to h}(i,j) + \boldsymbol{f}_r^{g\to h}(j)$, (11d)			
99.53	0.0484	96.03	0.00033
$\log \beta_{r,i,j}^{g\to h} = \hat{\boldsymbol{c}}^{g\to h}(i,j) + \gamma_0 + \gamma_g + \gamma_r + \gamma_{p(r)} + \boldsymbol{f}_0^{g\to h}(i,j) + \boldsymbol{f}_r^{g\to h}(j) + \boldsymbol{f}_{p(r)}^{g\to h}(j)$, (11e)			
99.55	0.0480	97.62	0.00031
$\log \beta_{r,i,j}^{g\to h} = \hat{\boldsymbol{c}}^{g\to h}(i,j) + \gamma_0 + \gamma_g + \gamma_r + \gamma_{p(r)} + \boldsymbol{f}_0^{g\to h}(i,j) + \boldsymbol{f}_r^{g\to h}(j) + \boldsymbol{f}_{p(r)}^{g\to h}(i,j)$, (11f)			
99.57	0.0482	97.46	0.00031

† MAE: Mean absolute error.

Supplementary Table S10: **Model comparison for estimating longitudinal, age-specific transmission flows.**

	Contribution from male sources to incidence			Median age of male sources			Median age of female sources			Counterfactual additional number of men suppressed			Counterfactual reduction in incidence in female		
	Round 10	Round 14	Round 18	Round 10	Round 14	Round 18	Round 10	Round 14	Round 18	Closing half the suppression gap	Closing the suppression gap	95-95-95 in men	Closing half the suppression gap	Closing the suppression gap	95-95-95 in men
**Central analysis**															
	57.9% [56.1-59.6]	61.4% [59.8-63.1]	62.8% [60.2-65.2]	29.0 [22.7-40.0]	30.6 [22.5-40.8]	33.7 [23.5-41.4]	25.0 [18.0-36.1]	26.3 [19.7-37.2]	26.0 [19.0-36.2]	75.1 [53.9-96.4]	150.2 [107.8-192.8]	172.6 [136.8-210.3]	25.2% [24.2-26.2]	50.6% [48.7-52.7]	58.4% [55.1-61.6]
**Sensitivity analyses**															
Using incidence rates estimated with LOESS regression															
	61.6% [59.7-63.6]	57.5% [55.5-59.4]	62.1% [60.4-63.9]	28.0 [22.0-39.1]	32.0 [23.0-43.2]	34.0 [23.9-42.0]	24.0 [18.0-35.1]	25.0 [19.0-37.0]	26.5 [19.0-37.1]	75.1 [53.9-96.4]	150.2 [107.8-192.8]	172.6 [136.8-210.3]	25.3% [24.2-26.5]	51.0% [48.7-53.5]	58.0% [53.8-61.8]
Using incidence rates estimated on a data subset to 28 continuously surveyed communities															
	58.0% [56.3-59.7]	62.3% [60.7-64.0]	64.2% [61.5-66.8]	29.8 [23.0-40.8]	31.0 [22.9-41.6]	34.0 [24.0-42.6]	25.0 [18.0-36.2]	27.0 [19.5-37.2]	26.7 [19.0-36.9]	75.1 [53.9-96.4]	150.2 [107.8-192.8]	172.6 [136.8-210.3]	25.5% [24.3-26.7]	51.4% [49.0-53.9]	56.7% [52.5-60.8]
Using non-refined infection time estimates															
	57.8% [56.1-59.4]	61.5% [59.8-63.1]	62.8% [60.2-65.2]	29.0 [22.6-40.2]	30.7 [22.3-41.1]	33.0 [23.7-41.8]	24.3 [18.0-36.0]	26.0 [19.4-37.4]	26.0 [19.0-36.6]	75.1 [53.9-96.4]	150.2 [107.8-192.8]	172.6 [136.8-210.3]	25.0% [24.0-26.1]	50.4% [48.3-52.5]	58.6% [55.2-61.9]
Without source-recipients pairs for which the source or recipient was sequenced after round 17															
	57.9% [56.2-59.6]	61.4% [59.8-63.1]	62.7% [60.1-65.2]	28.3 [22.5-39.8]	30.0 [22.3-40.6]	33.0 [23.0-41.3]	25.0 [18.2-36.5]	27.0 [20.0-37.6]	26.0 [20.0-36.4]	75.1 [53.9-96.4]	150.2 [107.8-192.8]	172.6 [136.8-210.3]	24.9% [23.9-26.0]	50.1% [48.0-52.3]	59.2% [55.7-62.7]
Without source-recipients pairs for which the source or recipient was sequenced after round 16															
	57.9% [56.2-59.6]	61.4% [59.7-63.0]	62.7% [60.2-65.1]	28.4 [22.6-39.7]	30.1 [22.4-40.5]	33.3 [23.9-41.0]	24.8 [18.0-36.2]	26.0 [20.0-37.4]	25.3 [19.7-35.4]	75.1 [53.9-96.4]	150.2 [107.8-192.8]	172.6 [136.8-210.3]	24.9% [23.7-26.1]	50.2% [47.7-52.7]	58.9% [55.1-62.8]
Without source-recipients pairs for which the source or recipient was sequenced after round 15															
	57.9% [56.2-59.6]	61.4% [59.8-63.1]	62.7% [60.2-65.2]	28.4 [22.5-39.5]	30.0 [22.2-40.5]	33.0 [24.0-41.1]	25.0 [18.0-36.8]	26.4 [19.8-38.0]	26.0 [19.8-36.0]	75.1 [53.9-96.4]	150.2 [107.8-192.8]	172.6 [136.8-210.3]	24.9% [23.6-26.2]	50.1% [47.4-52.7]	58.9% [54.8-63.2]
Using a bootstrap sample of the source-recipient pairs (first draw)															
	57.9% [56.2-59.5]	61.4% [59.8-63.1]	62.7% [60.2-65.2]	28.6 [22.2-40.6]	30.5 [22.0-41.5]	34.3 [23.2-42.0]	23.8 [17.0-35.4]	25.9 [19.0-37.5]	25.0 [18.4-34.6]	75.1 [53.9-96.4]	150.2 [107.8-192.8]	172.6 [136.8-210.3]	25.5% [24.6-26.6]	51.4% [49.4-53.6]	57.1% [53.7-60.5]
Using a bootstrap sample of the source-recipient pairs (second draw)															
	57.8% [56.1-59.5]	61.4% [59.8-63.1]	62.7% [60.2-65.2]	28.3 [22.6-40.1]	30.2 [22.4-40.9]	33.0 [24.0-41.0]	24.0 [17.8-34.8]	25.1 [19.1-36.6]	25.0 [19.0-34.6]	75.1 [53.9-96.4]	150.2 [107.8-192.8]	172.6 [136.8-210.3]	24.9% [23.9-25.9]	50.1% [48.0-52.2]	59.2% [56.1-62.5]
Using a bootstrap sample of the source-recipient pairs (third draw)															
	57.9% [56.2-59.6]	61.4% [59.8-63.1]	62.7% [60.2-65.2]	28.0 [22.2-40.4]	30.0 [22.0-41.4]	33.0 [24.2-41.8]	24.3 [18.0-34.7]	26.0 [19.6-36.5]	25.6 [19.1-35.7]	75.1 [53.9-96.4]	150.2 [107.8-192.8]	172.6 [136.8-210.3]	25.0% [24.0-26.0]	50.4% [48.4-52.4]	58.3% [55.2-61.5]

Supplementary Table S11: **Sensitivity analyses.**

medRxiv preprint doi: https://doi.org/10.1101/2023.03.16.23287351; this version posted March 20, 2023. The copyright holder for this preprint (which was not certified by peer review) is the author/funder, who has granted medRxiv a license to display the preprint in perpetuity. It is made available under a CC-BY 4.0 International license.

Contribution from male sources to incidence			Median age of male sources			Median age of female sources			Counterfactual additional number of men suppressed			Counterfactual reduction in incidence in female		
Round 10	Round 14	Round 18	Round 10	Round 14	Round 18	Round 10	Round 14	Round 18	Closing half the suppression gap	Closing the suppression gap	95-95-95 in men	Closing half the suppression gap	Closing the suppression gap	95-95-95 in men
Assuming an alternative form of the transmission rate (11a))														
57.9% [56.2-59.6]	61.4% [59.7-63.0]	62.7% [60.2-65.1]	29.0 [22.9-40.0]	30.9 [22.7-40.8]	33.0 [23.0-42.0]	25.0 [18.0-36.1]	26.4 [19.9-37.2]	25.8 [19.0-36.6]	75.1 [53.9-96.4]	150.2 [107.8-192.8]	172.6 [136.8-210.3]	25.0% [24.2-25.7]	50.3% [48.8-51.9]	58.8% [56.1-61.5]
Assuming an alternative form of the transmission rate (11b))														
57.9% [56.2-59.5]	61.4% [59.8-63.1]	62.7% [60.2-65.2]	29.0 [22.8-40.0]	30.8 [22.7-40.7]	33.0 [23.0-41.7]	25.0 [18.0-36.0]	26.3 [19.8-37.2]	26.0 [19.0-36.5]	75.1 [53.9-96.4]	150.2 [107.8-192.8]	172.6 [136.8-210.3]	25.0% [24.1-25.9]	50.4% [48.5-52.3]	58.9% [55.6-62.0]
Assuming an alternative form of the transmission rate (11c))														
60.3% [59.3-61.2]	60.8% [59.9-61.7]	64.4% [63.1-66.0]	29.3 [22.8-44.6]	32.1 [23.0-48.5]	35.0 [24.2-43.0]	24.5 [17.9-36.2]	26.0 [19.4-37.3]	26.0 [19.2-36.6]	75.1 [53.9-96.4]	150.2 [107.8-192.8]	172.6 [136.8-210.3]	25.6% [24.8-26.4]	51.7% [50.1-53.4]	55.8% [52.9-59.7]
Assuming an alternative form of the transmission rate (11d))														
60.5% [59.6-61.5]	60.7% [59.8-61.5]	63.9% [62.5-65.2]	30.1 [23.0-42.3]	32.4 [23.0-46.2]	33.7 [23.0-46.3]	24.7 [18.0-36.3]	26.1 [19.5-37.4]	25.2 [18.9-36.6]	75.1 [53.9-96.4]	150.2 [107.8-192.8]	172.6 [136.8-210.3]	25.0% [24.3-25.6]	50.6% [49.1-52.0]	56.7% [53.7-60.2]
Assuming an alternative form of the transmission rate (11e))														
60.4% [59.4-61.3]	60.7% [59.9-61.6]	64.0% [62.7-65.4]	30.0 [23.0-43.9]	32.6 [23.0-48.2]	33.8 [23.2-44.6]	24.7 [18.0-36.4]	26.1 [19.5-37.5]	26.0 [19.0-36.7]	75.1 [53.9-96.4]	150.2 [107.8-192.8]	172.6 [136.8-210.3]	25.0% [24.2-25.9]	50.6% [48.9-52.5]	56.9% [53.7-60.1]
Assuming an alternative form of the transmission rate (11f))														
57.8% [56.1-59.5]	61.4% [59.8-63.1]	62.8% [60.2-65.2]	29.0 [22.9-40.0]	30.9 [22.8-40.7]	33.0 [23.0-41.8]	25.0 [18.0-35.9]	26.2 [19.8-37.2]	25.7 [19.0-36.5]	75.1 [53.9-96.4]	150.2 [107.8-192.8]	172.6 [136.8-210.3]	25.0% [24.2-25.8]	50.3% [48.8-51.9]	58.9% [56.2-61.6]
Assuming the same suppression rate in non-participants as in participants														
57.9% [56.2-59.5]	61.4% [59.8-63.1]	62.7% [60.1-65.2]	29.0 [22.7-40.1]	30.6 [22.4-40.6]	33.0 [23.4-41.3]	25.0 [18.0-36.1]	26.3 [19.7-37.2]	26.0 [19.0-36.3]	71.7 [54.6-89.5]	143.3 [109.3-179.0]	143.5 [114.1-175.7]	26.7% [26.1-27.2]	53.5% [52.5-54.6]	52.3% [47.4-56.9]
Assuming that non-participants are not suppressed														
57.9% [56.2-59.5]	61.4% [59.8-63.1]	62.7% [60.2-65.1]	28.9 [22.7-40.0]	30.6 [22.4-40.9]	34.1 [24.0-41.9]	25.0 [18.0-36.0]	26.3 [19.8-37.2]	26.2 [19.0-37.8]	254.7 [232.7-275.5]	329.9 [300.0-358.6]	351.9 [333.4-372.5]	52.4% [50.1-54.6]	68.3% [65.9-70.5]	74.6% [73.5-75.8]
Assuming that prevalence in non-participants is 25% higher than in participants														
57.9% [56.2-59.6]	61.5% [59.8-63.1]	62.6% [60.2-65.1]	29.0 [22.7-40.0]	30.5 [22.4-40.7]	33.7 [23.5-41.4]	25.0 [18.0-36.2]	26.4 [19.7-37.3]	26.0 [19.0-36.2]	81.9 [58.9-105.1]	163.9 [117.7-210.1]	189.3 [150.4-230.4]	25.2% [24.2-26.2]	50.7% [48.7-52.8]	58.4% [55.1-61.6]
Assuming that prevalence in men non-participants is 25% higher than in men participants														
58.1% [56.4-59.8]	61.7% [60.1-63.3]	62.9% [60.4-65.4]	29.0 [22.7-40.1]	30.6 [22.5-40.8]	33.7 [23.5-41.4]	25.0 [18.0-36.1]	26.3 [19.7-37.2]	26.0 [19.0-36.2]	81.9 [58.9-105.1]	163.9 [117.7-210.1]	189.3 [150.4-230.4]	25.2% [24.2-26.2]	50.7% [48.7-52.7]	58.3% [55.1-61.6]
Assuming that prevalence in women non-participants is 25% higher than in women participants														
57.7% [55.9-59.4]	61.2% [59.6-62.8]	62.5% [60.0-64.9]	29.0 [22.7-40.0]	30.6 [22.5-40.8]	33.7 [23.5-41.4]	25.0 [18.0-36.3]	26.4 [19.8-37.3]	26.0 [19.0-36.2]	75.1 [53.9-96.4]	150.2 [107.8-192.8]	172.6 [136.8-210.3]	25.2% [24.2-26.2]	50.7% [48.7-52.7]	58.4% [55.1-61.7]
Defining viral suppression as a viral load measurement below 200 copies/mL plasma blood														
57.9% [56.1-59.6]	61.4% [59.8-63.1]	62.7% [60.2-65.2]	29.0 [22.7-40.1]	30.7 [22.5-40.8]	33.3 [23.4-41.3]	25.0 [18.0-36.2]	26.7 [19.8-37.3]	26.0 [19.4-36.5]	73.2 [51.7-94.5]	146.4 [103.5-189.1]	197.2 [161.7-234.6]	22.9% [21.8-24.0]	46.1% [44.0-48.5]	61.6% [58.3-64.7]

Supplementary Table S11: **Sensitivity analyses (continued).**

medRxiv preprint doi: https://doi.org/10.1101/2023.03.16.23287351; this version posted March 20, 2023. The copyright holder for this preprint (which was not certified by peer review) is the author/funder, who has granted medRxiv a license to display the preprint in perpetuity. It is made available under a CC-BY 4.0 International license .

medRxiv preprint doi: https://doi.org/10.1101/2023.03.16.23287351; this version posted March 20, 2023. The copyright holder for this preprint (which was not certified by peer review) is the author/funder, who has granted medRxiv a license to display the preprint in perpetuity.
It is made available under a CC-BY 4.0 International license .

S1 Supplementary Material

medRxiv preprint doi: https://doi.org/10.1101/2023.03.16.23287351; this version posted March 20, 2023. The copyright holder for this preprint (which was not certified by peer review) is the author/funder, who has granted medRxiv a license to display the preprint in perpetuity.
It is made available under a CC-BY 4.0 International license .

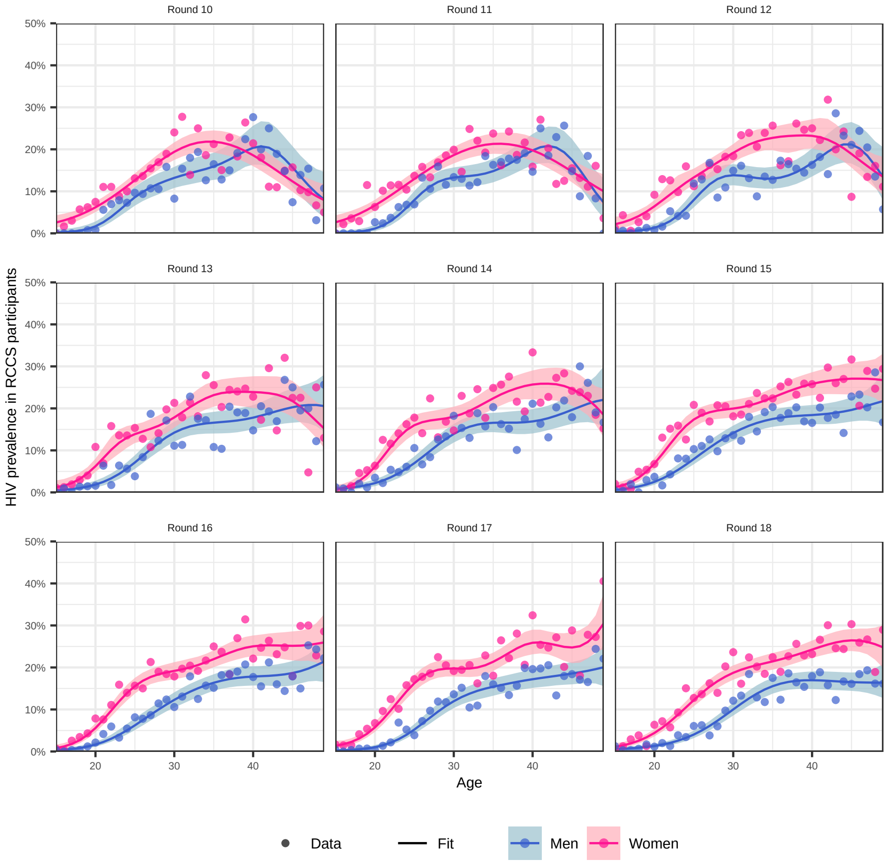

Supplementary Fig. S1: **HIV prevalence estimates by age, gender, and time.** HIV positivity rates in RCCS study participants are shown by 1-year age band (x-axis), for each survey round (panel) and gender (colour). Posterior median estimates of HIV prevalence are shown as a line, and 95% credible intervals as a ribbon. Crude estimates are shown with dots. The timeline of the survey rounds is shown in Fig. 1b.

medRxiv preprint doi: https://doi.org/10.1101/2023.03.16.23287351; this version posted March 20, 2023. The copyright holder for this preprint (which was not certified by peer review) is the author/funder, who has granted medRxiv a license to display the preprint in perpetuity.
It is made available under a CC-BY 4.0 International license .

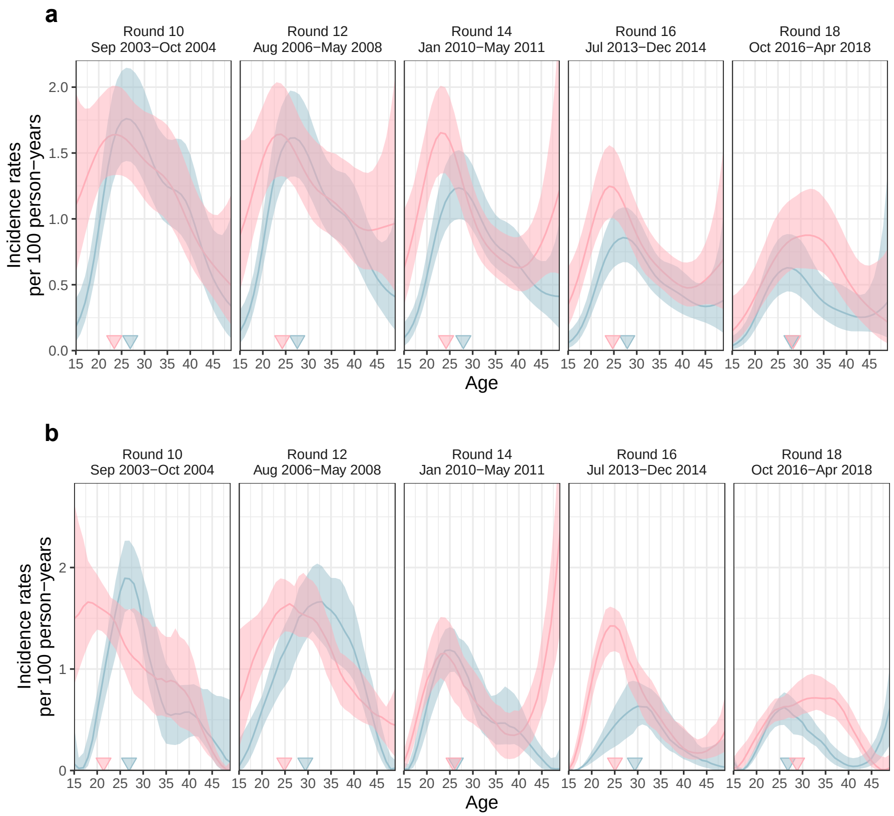

Supplementary Fig. S2: **Comparison of incidence rate estimates under an individual-level additive effects Poisson regression model and a population-level LOESS model with independent age effects in each survey round.** (**a**) Mean and 95% uncertainty ranges of longitudinal age-specific incidence rates obtained with the individual-level additive effects Poisson regression model used in the central analysis (**b**) Same using a population-level LOESS model with independent age effects in each survey round.

medRxiv preprint doi: https://doi.org/10.1101/2023.03.16.23287351; this version posted March 20, 2023. The copyright holder for this preprint (which was not certified by peer review) is the author/funder, who has granted medRxiv a license to display the preprint in perpetuity.
It is made available under a CC-BY 4.0 International license .

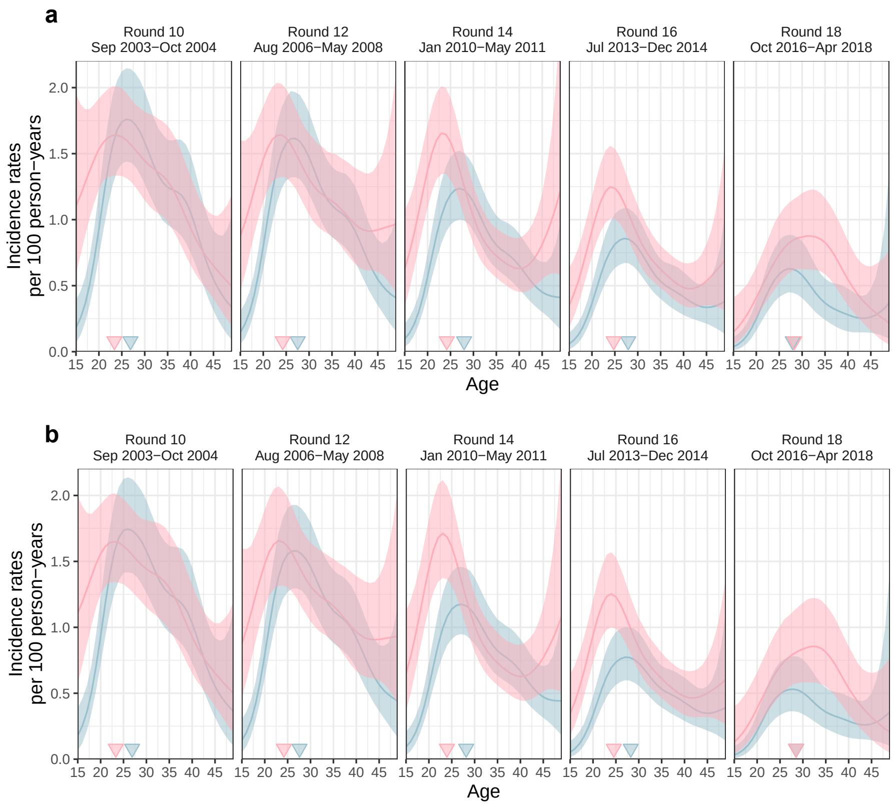

Supplementary Fig. S3: **Comparison of incidence rate estimated on data containing all communities and data subset to 28 continuously surveyed communities** (**a**) Mean and 95% uncertainty ranges of longitudinal age-specific incidence rates estimated on data from all communities surveyed (**b**) Same using data subset to 28 continuously surveyed communities.

medRxiv preprint doi: https://doi.org/10.1101/2023.03.16.23287351; this version posted March 20, 2023. The copyright holder for this preprint (which was not certified by peer review) is the author/funder, who has granted medRxiv a license to display the preprint in perpetuity.
It is made available under a CC-BY 4.0 International license .

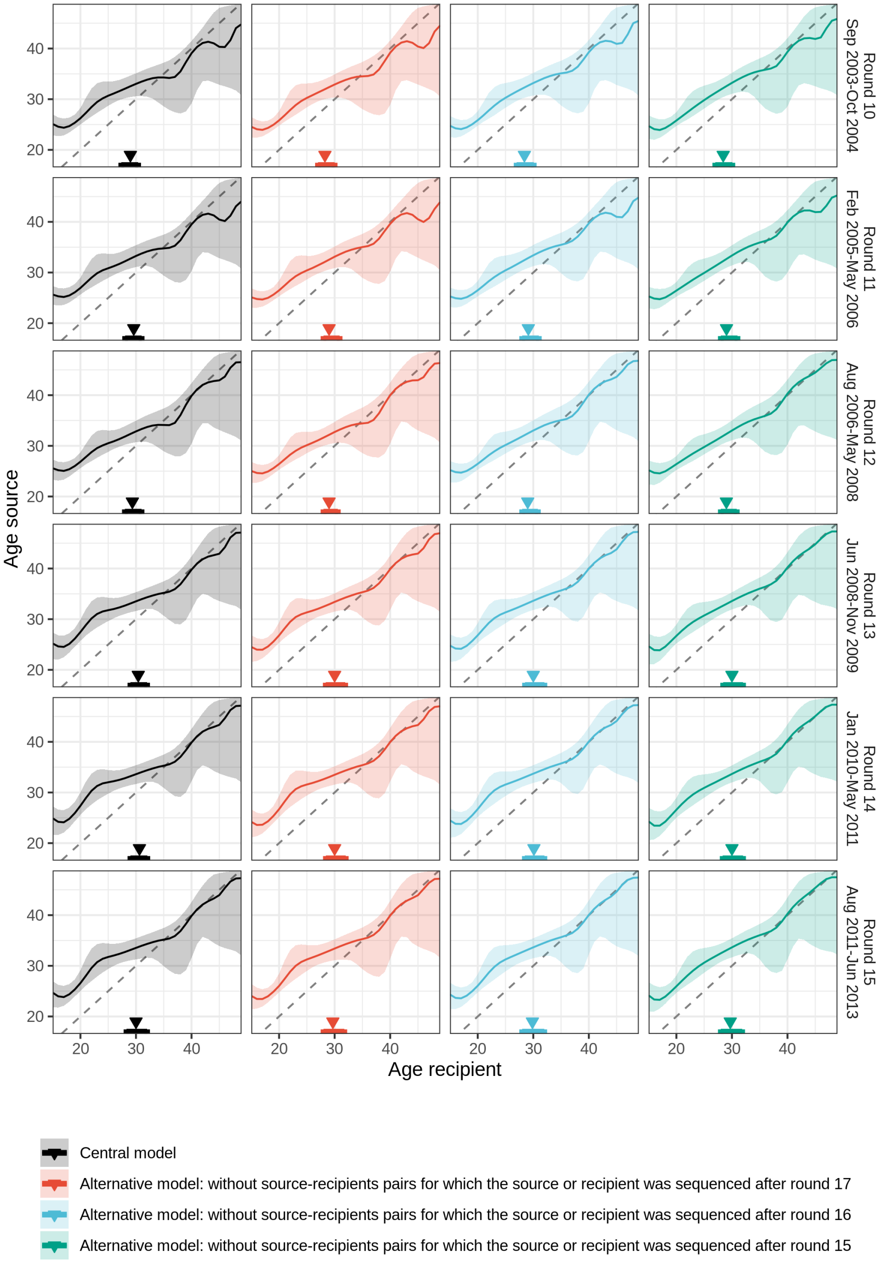

Supplementary Fig. S4: **Sensitivity in estimating the age of transmitting partners to right censoring of likely transmission pairs.** Posterior median (line) and 95% credible interval (ribbon) of the age of male transmitting partners by the age of the infected female (x-axis) by survey round (row facet) for the central and sensitivity analyses (column facet). Median and 95% credible interval of the age of male transmitting partners across the age of the infected female is indicated with a triangle and an error bar.
